# Significant Improvement of Optoelectronic and Photovoltaic Properties by Incorporating Thiophene in a Solution-Processable D–A–D Modular Chromophore

**DOI:** 10.3390/molecules201219798

**Published:** 2015-12-04

**Authors:** Aaron M. Raynor, Akhil Gupta, Christopher M. Plummer, Sam L. Jackson, Ante Bilic, Hemlata Patil, Prashant Sonar, Sheshanath V. Bhosale

**Affiliations:** 1 School of Applied Sciences, The Royal Melbourne Institute of Technology (RMIT) University, GPO Box 2476, Melbourne Victoria 3001, Australia; aaron.raynor@rmit.edu.au (A.M.R.); chris.plummer@rmit.edu.au (C.M.P.); sam.jackson@rmit.edu.au (S.L.J.); hemlatap2@gmail.com (H.P.); 2 Medicinal Chemistry, Monash Institute of Pharmaceutical Sciences, Monash University, Parkville Victoria 3052, Australia; 3 Virtual Nanoscience Lab, Commonwealth Scientific and Industrial Research Organization (CSIRO) Manufacturing, Parkville Victoria 3052, Australia; ante.bilic@csiro.au; 4 School of Chemistry, Physics and Mechanical Engineering, Queensland University of Technology (QUT), GPO Box 2434, Brisbane QLD 4001, Australia

**Keywords:** solution-processable, bulk-heterojunction devices, donor–acceptor–donor, triphenylamine, thiophene, 1,4-phenylenediacetonitrile

## Abstract

Through the incorporation of a thiophene functionality, a novel solution-processable small organic chromophore was designed, synthesized and characterized for application in bulk-heterojunction solar cells. The new chromophore, (2*Z*,2′*Z*)-2,2′-(1,4-phenylene)bis(3-(5-(4-(diphenylamino)phenyl)thiophen-2-yl)acrylonitrile) (coded as **AS2**), was based on a donor–acceptor–donor (D–A–D) module where a simple triphenylamine unit served as an electron donor, 1,4-phenylenediacetonitrile as an electron acceptor, and a thiophene ring as the π-bridge embedded between the donor and acceptor functionalities. **AS2** was isolated as brick-red, needle-shaped crystals, and was fully characterized by ^1^H- and ^13^C-NMR, IR, mass spectrometry and single crystal X-ray diffraction. The optoelectronic and photovoltaic properties of **AS2** were compared with those of a structural analogue, (2*Z*,2′*Z*)-2,2′-(1,4-phenylene)bis(3-(4-(diphenylamino)phenyl)-acrylonitrile) (**AS1**). Benefiting from the covalent thiophene bridges, compared to **AS1** thin solid film, the **AS2** film showed: (1) an enhancement of light-harvesting ability by 20%; (2) an increase in wavelength of the longest wavelength absorption maximum (497 nm *vs.* 470 nm) and (3) a narrower optical band-gap (1.93 eV *vs.* 2.17 eV). Studies on the photovoltaic properties revealed that the best **AS2**-[6,6]-phenyl-C_61_-butyric acid methyl ester (PC_61_BM)-based device showed an impressive enhanced power conversion efficiency of 4.10%, an approx. 3-fold increase with respect to the efficiency of the best **AS1**-based device (1.23%). These results clearly indicated that embodiment of thiophene functionality extended the molecular conjugation, thus enhancing the light-harvesting ability and short-circuit current density, while further improving the bulk-heterojunction device performance. To our knowledge, **AS2** is the first example in the literature where a thiophene unit has been used in conjunction with a 1,4-phenylenediacetonitrile accepting functionality to extend the π-conjugation in a given D–A–D motif for bulk-heterojunction solar cell applications.

## 1. Introduction

The development of renewable energy technologies is pivotal for accommodating the ever increasing energy demands of the modern society. Such technologies are also important for lowering environmental pollution and greenhouse gas emissions. Towards this objective, many approaches to harvest solar energy have been investigated. The fabrication of bulk-heterojunction (BHJ) devices is one such promising strategy that has attracted considerable attention over the past two decades due to their advantages of being lightweight, low cost and their flexibility in large-area applications [1,2,3,4,5,6,7]. Such devices are comprised of an interpenetrating network of organic donor and acceptor domains that is formed during their fabrication via solution processing. Conventionally, semiconducting donor polymers such as poly(3-hexylthiophene) (P3HT) and acceptors such as soluble fullerene derivatives, PC_61_BM and its C_71_ analogue (PC_71_BM), have been used to obtain a deeper understanding of device design and morphology [8,9,10,11,12,13]. Apart from archetypal P3HT, conjugated polymers have also been developed and significant progress has been attained with promising BHJ architecture. Power conversion efficiency (PCE) values above 10% has been reported with such polymeric donors [14,15,16,17]. In the interim, solution-processed small molecular donor-based BHJ devices have also aroused interest, mainly due to their advantages of well-defined chemical structure, convenient purification methods, such as simple column chromatography, and monodisperse molecular weight [18,19,20,21,22,23]. These advantages allow and encourage researchers to exert efforts for the design and development of small molecular donors. Recently, immense efforts have been dedicated to developing small molecular-based solution-processable organic solar cells [1,21] and so far, the highest PCE of 9.95% was achieved by Kan *et al.* [24] which is analogous to those of the polymer-based solution-processable devices. Thus, in view of such reports and the fact that BHJ devices incorporating small molecular donors can compete with polymer-based devices, there is an overwhelming interest in developing small molecular donors.

Recent years have seen a dramatic surge not only in terms of device efficiency using small molecular donors but also in their design and efficient synthetic development. A variety of small molecule donor materials based on donor–acceptor (D–A) combinations such as D–A–D [25,26,27], A–D–A [28,29], D–π–A [18,19,30] and star-shaped architectures [31] have been reported. The D–A–D design in particular is one of the most promising and successful modules based on which various donor and acceptor units have been explored for high-performance solution-processable photovoltaic devices. A finite number of central accepting units, such as naphthalene diimide [25], diketopyrrolopyrrole [27], 2-pyran-4-ylidenemalononitrile [32] and thiazolothiazole [33] have been reported to suit the D–A–D module. Not only that the availability and selection of such accepting blocks is limited, it is furthermore imperative that the target chromophore must possess a low optical band gap, broad absorption profile, high mobility and appropriately tuned highest occupied molecular orbital (HOMO) and lowest unoccupied molecular orbital (LUMO) energy levels using such blocks. Such requirements do possess a challenge for an organic chemist who must consider such factors while designing a new chromophore based on the D–A–D module. Therefore, it is not surprising that there exists an enormous scope for the design and development of new light-harvesting materials based on the challenging D–A–D module and is an aspiration for most of the researchers.

**Figure 1:**
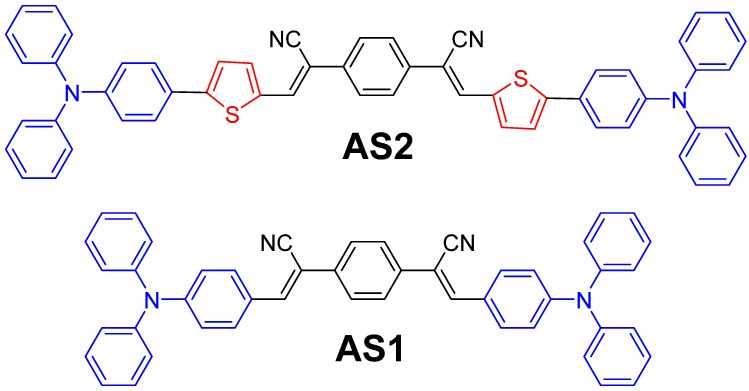
Molecular structures of the newly designed **AS2** and reference **AS1** materials investigated in this study.

In our own studies of small molecule chromophores and charge transport materials based on a variety of D–A combinations, we have reported examples of successful solution-processable BHJ photovoltaic devices [18,19,25,34,35,36,37]. Furthermore, we are highly interested to extend our efforts on the design and development of new chromophores that are inspired by the D–A–D module. In this study, we report the design, facile synthesis and characterization of the optoelectronic and photovoltaic properties of two small organic chromophores, **AS2** and **AS1**, (shown in Figure 1), and their direct comparison. Both materials are based on a D–A–D structural motif where a triphenyl-amine functionality has been chosen as a common donor at both ends of the central acceptor unit, 1,4-phenylenediacetonitrile (PDA), so as to get symmetrical **AS2** and **AS1**. **AS2** is a structural analogue of **AS1** where a thiophene ring has been introduced between the donor and acceptor functionalities in order to vary the optoelectronic and photovoltaic properties (Figure 1). When compared with the commonly used accepting groups, such as dicyanovinylidene, aromatizable cyanopyridone, indenedione or oxoindenemalononitrile [38,39], PDA can be an acceptor of choice for extended π-conjugation over the whole molecular backbone, mainly due to its bidentate nature. It is notable to mention that the use of the PDA unit for electroluminescent conjugated polymers has been reported in the literature [40,41] as well as one- and two-photon spectroscopy studies [42,43,44,45,46]. However, its suitability as an acceptor in small molecular chromophores, particularly D–A–D modular, for BHJ applications is still unknown [4,21]. This provides an encouragement and some strong incentive for its investigation. Owing to the exploration of PDA acceptor unit in the D–A–D module, this study continues our search for the generation of new organic chromophores for BHJ applications.

## 2. Results and Discussion

### 2.1. Design Strategy, Synthesis

The materials **AS2** and **AS1** were synthesized via the Knoevenagel condensation of appropriate aldehydes with active methylene groups of the PDA acceptor unit and their chemical structures were confirmed by ^1^H- and ^13^C-NMR, mass spectrometry, and, where possible (**AS2** only), by single crystal X-ray diffraction (XRD). The synthetic methodology for synthesizing **AS1** was similar to an old literature report [42], albeit dissimilar base and solvent were used to deal with a homogeneous reaction solution. Knoevenagel condensation reaction of an aldehyde with active methylene group is an efficient way of generating a double bond between a π-bridge and acceptor functionality. The use of such chemistry is a common strategy to generate organic sensitizers for dye-sensitized solar cells [47]. However, the use of same strategy to develop small molecular chromophores for BHJ applications is still limited [4,21]. Herein, not only we are demonstrating the Knoevenagel condensation reaction of PDA acceptor unit but also the fabrication of solution-processable BHJ devices incorporating a fullerene acceptor (PC_61_BM) and either **AS2** or **AS1** as a donor component (Figure 1). To the best of our knowledge, this is the first time PDA has been used to generate D–A–D modular small molecular chromophores for BHJ applications. Initial screening of the BHJ devices revealed that greater PCE was achieved for **AS2** (4.10% for **AS2** compared with 1.23% for **AS1**), as confirmed by the increased short-circuit current density (8.01 mA·cm^−2^ for **AS2** and 3.15 mA·cm^−2^ for **AS1**), under simulated AM 1.5 illumination (100 mW·cm^−2^).

**Scheme 1:**
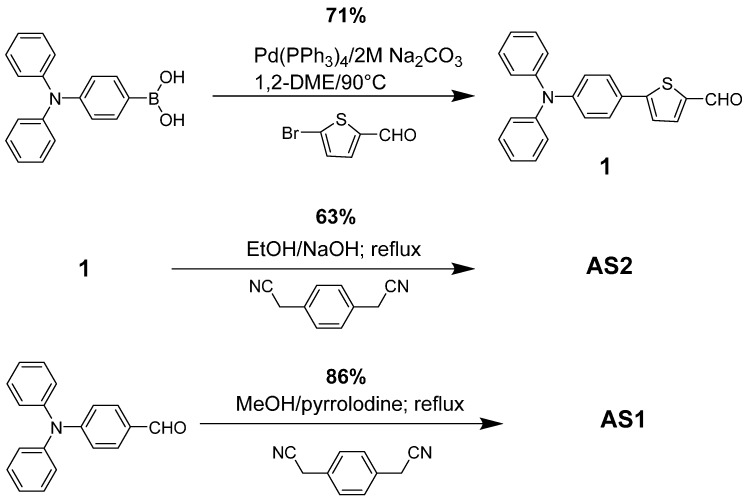
Reaction scheme for the synthesis of **AS2** and **AS1**.

Both materials were based on the D–A–D module and the central acceptor moiety was directly linked to the terminal donor functionalities in order to create a conjugated structure. The development of these target materials incorporates the use of two identical donor units (triphenylamine) on each side of the central core, resulting in symmetrical chromophores. Insertion of a planar, conjugated functionality, such as thiophene in **AS2**, between the donor and acceptor components of a target material can provide greater absorption over visible light spectrum when compared with otherwise structurally similar compounds [38,48]. Moreover, the selection of thiophene over highly aromatic, conjugating functionalities, such as phenyl, was based on the earlier work reported by Würthner *et al.* [49] and Gupta *et al.* [50] where it has been demonstrated that replacement of a phenyl group with thiophene can provide significant spectral red-shifts and is advantageous for superior charge delocalization. As a result, **AS2** is deemed to exhibit a large red shift of lambda maximum when compared with the reference compound **AS1**. Both of the materials were synthesized per the reaction shown in Scheme 1 and were purified by simple column chromatography. Brick-red, needle-shaped crystals, suitable for single crystal XRD, were prepared by diffusing methanol into a dichloromethane solution of **AS2**, over approximately three days. However, no crystal growth was observed for **AS1**. Both materials were synthesized in moderate to high yields (**AS2** = 63% and **AS1** = 86%) and were highly soluble in a variety of common organic solvents, for example chlorobenzene, chloroform, and toluene. The solubility of organic p-type materials is paramount for fabricating solution-processable BHJ devices and both the materials fulfill this criterion. In fact, the solubility of **AS2** was found to be higher by 50% *w*/*v* when compared with **AS1**.

The precursor aldehyde **1** of **AS2** was also crystallized by diffusion of methanol into chloroform to obtain yellow, needle-shaped crystals. Diffraction measurements were performed at 200 K on a Bruker Apex II CCD diffractometer using Mo Kα radiation(λ = 0.71073 Å) The structure were solved using dual space methods using the program SHELX-2014/7 [51] using the Olex2 1.2 GUI [52], with anisotropic thermal parameters for all non-hydrogen atoms. All non-hydrogen atoms were refined anisotropically by full-matric least-squares methods SHELX-2014/7. Molecular drawings were obtained using Mercury [53]. The utility of **1** for dye-sensitized solar cells has been reported [54], however, its use to generate small molecular chromophores for BHJ applications is seldom reported [4,21]. This state of affairs encourages us and provides a strong incentive to report its synthesis and crystal growth strategy.

**Figure 2:**
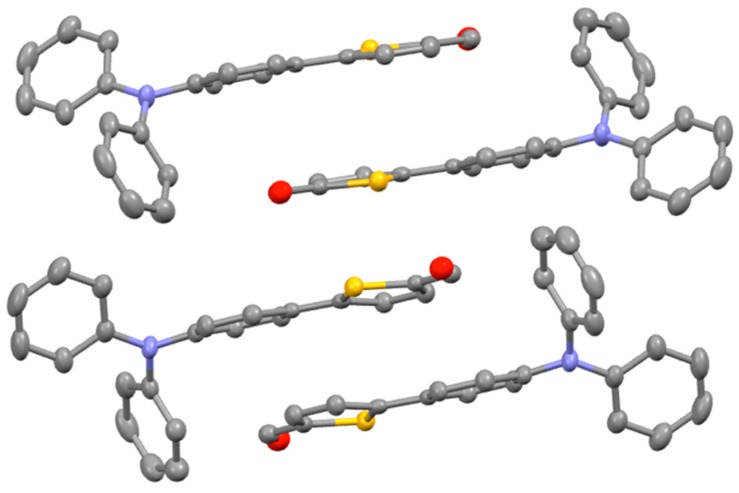
Packing of **1** along the *b* axis.

The compound **1** was crystallized in the monoclinic space group (P 2_1_/c) with four asymmetric units in one cell. The thiophene group and the adjacent phenyl groups are planar with pendant phenyl groups displaced around nitrogen. The packing consist of a two-fold screw axis with centers of inversion between sulfur molecules as well as a glide plane perpendicular to the thiophene plane. The packing is dominated by π-π face-to-face stacking between the thiophene and phenyl groups. The pendant phenyl groups are stabilized by π-π edge-to-face stacking with distances in the range 2.771–3.283 Å as shown in Figure 2.

**Figure 3:**
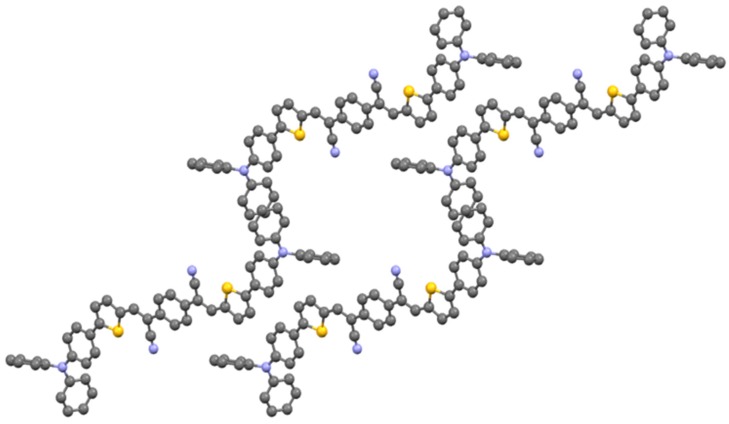
Packing of **AS2** along the *c* axis.

**AS2** co-crystallizes with chloroform in the triclinic space group (P-1) with a center of symmetry around the central phenyl group. The packing is dominated by face-to-face π-π stacking between central phenyls and thiophenes, and by edge-to-face π‑π‑stacking between pendant phenyl groups with the distance 2.772 Å as shown in Figure 3. The details of packing structure, formula, crystal size of **1** and **AS2** are described in Table 1.

**Table 1:** Details of crystal data and structure refinement parameters of **1** and **AS2**.

Identification Code	CCDC: 1420377 (1)	CCDC: 1420378 (AS2)
Empirical formula	C_23_H_17_NOS	C_31_H_22_OS
Formula weight	355.43	442.55
Temperature/K	200(2)	200(2)
Crystal system	monoclinic	monoclinic
Space group	P2_1_/c	P2_1_
a/Å	19.916(3)	5.5812(7)
b/Å	6.6680(9)	47.120(6)
c/Å	13.4336(16)	9.2435(11)
α/°	90	90
β/°	96.613(3)	103.343(3)
γ/°	90	90
Volume/Å^3^	1772.1(4)	2365.3(5)
Z	4	4
ρ_calc_g/cm^3^	1.332	1.243
μ/mm^−1^	0.194	0.158
F(000)	744.0	928.0
Crystal size/mm^3^	0.292 × 0.076 × 0.063	0.667 × 0.137 × 0.087
Radiation	MoKα (λ = 0.71073)	MoKα (λ = 0.71073)
2Θ range for data collection/	4.118 to 48.588	3.458 to 67.842
Index ranges	−22 ≤ h ≤ 23, −7 ≤ k ≤ 7, −15 ≤ l ≤ 15	−8 ≤ h ≤ 8, −73 ≤ k ≤ 73, −14 ≤ l ≤ 12
Reflections collected	15255	83,300
Independent reflections	2883 [R_int_ = 0.0707, R_sigma_ = 0.0447]	19,180 [R_int_ = 0.0616, R_sigma_ = 0.0596]
Data/restraints/parameters	2883/0/235	19180/1/595
Goodness-of-fit on F^2^	0.949	1.028
Final R indexes [I ≥ 2σ (I)]	R_1_ = 0.0412, wR_2_ = 0.1095	R_1_ = 0.0659, wR_2_ = 0.1531
Final R indexes [all data]	R_1_ = 0.0720, wR_2_ = 0.1274	R_1_ = 0.0972, wR_2_ = 0.1684
Largest diff. peak/hole/e Å^−3^	0.17/−0.26	0.29/−0.39

### 2.2. Optoelectronic Properties

**Figure 4:**
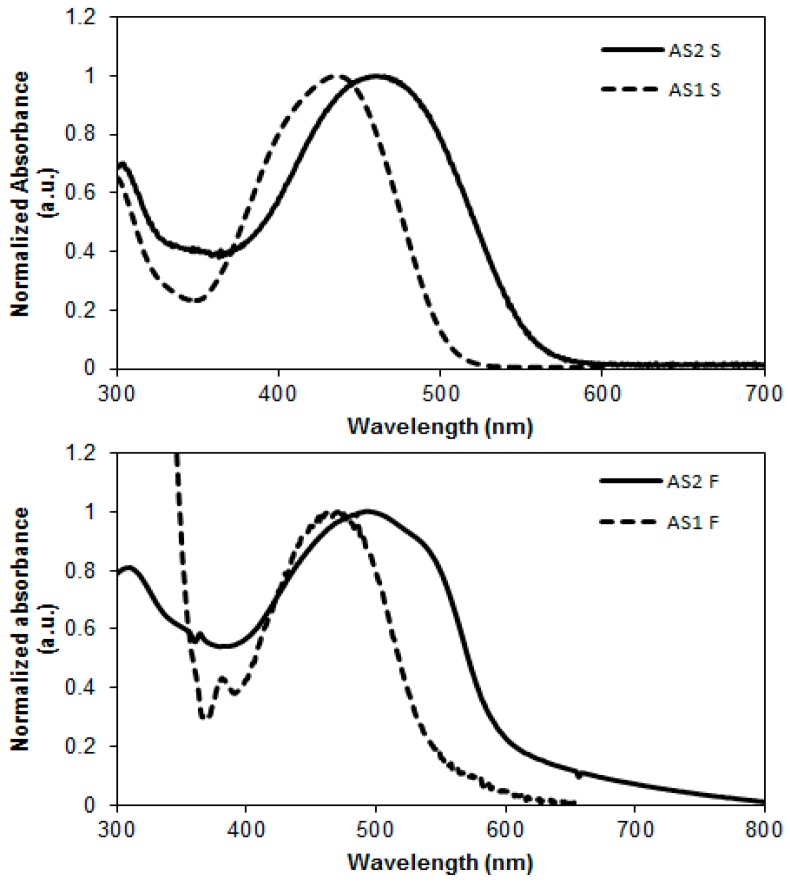
Normalized absorption spectra of compounds **AS2** and **AS1** in CHCl_3_ solutions (**upper**) and for pristine as-casted films (**lower**); (films of **AS2** and **AS1** were spin-coated at 2000 rpm for 1 min to give a film thickness of ~70 nm).

The optical properties of **AS2** and **AS1** were investigated by measuring their ultraviolet–visible (UV–Vis) absorption spectra in chloroform solution and in pristine spin-cast films (Figure 4). The longest wavelength absorption maximum (λ_max_) exhibited by **AS2** in solution form was at 459 nm which was red-shifted by 23 nm when compared with the solution λ_max_ of **AS1**. Both the absorption maximum and extinction coefficient (**AS2** = 59,000 M^−1^·cm^−1^; **AS1** = 49,000 M^−1^·cm^−1^) increased with the insertion of thiophene functionality. With the insertion of thiophene ring we found enhancement to the peak molar absorptivity of >20% of **AS2** compared with **AS1**. This enhanced profile allows a larger amount of the solar spectrum to be absorbed, thus exhibiting greater intramolecular charge transfer (ICT) transition. We observed a similar bathochromic absorption shift in the thin film spectrum of **AS2** compared with that of **AS1** (Figure 4). The strong red-shift is attributed to the extended π-conjugation within the molecular backbone of **AS2** that became possible with the insertion of thiophene functionality. This type of control over the absorption profile through the insertion of a strongly conjugated unit can help to fine tune optical energy levels, to enhance light harvesting and BHJ device performance.

**Figure 5:**
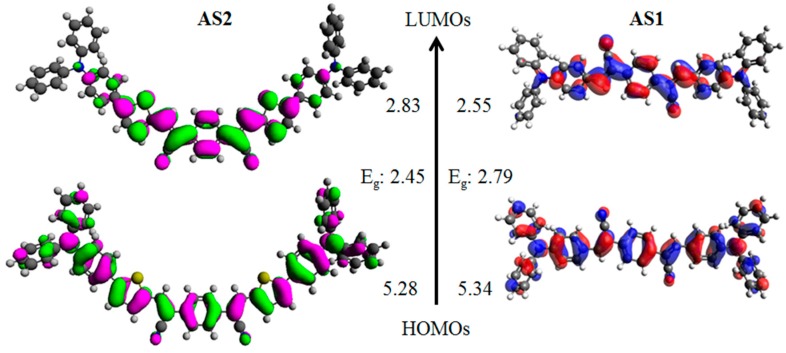
Orbital density distribution for the frontier molecular orbitals of **AS2** and **AS1**. DFT calculations were performed using the Gaussian 09 suite of programs and the B3LYP/6-311 + G(d,p)//B3LYP/6-31G(d) level of theory. Theoretical HOMO/LUMO energy levels and band-gaps (*vs*. Vac scale) are also shown.

**Figure 6:**
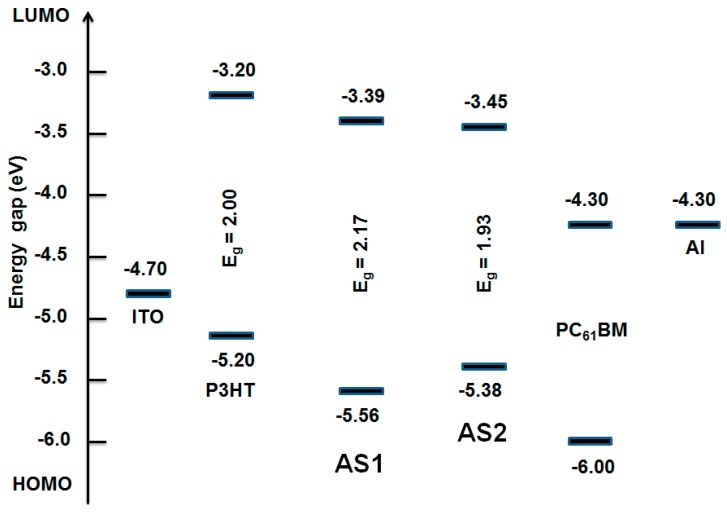
Energy level diagram depicting the band gaps of AS2 and AS1 in comparison with P3HT and PC_61_BM. Experimental estimation of the HOMO energies was carried out using PESA and the LUMO energies were calculated by adding the optical band gap (film spectra) to the HOMO values.

Density functional theory (DFT) calculations using the Gaussian 09 suite of programs [55] and the B3LYP/6-311 + G(d,p)//B3LYP/6-31G(d) level of theory indicated that the HOMO orbital densities of both **AS2** and **AS1** have a major distribution over the whole molecular backbone and the LUMO densities were delocalized through the central acceptor functionality and adjacent rings (Figure 5). Inserted thiophene rings in case of **AS2** can accommodate LUMO density with almost equal contribution as the central PDA, thus sparing the adjoining phenyl rings of the donor triphenylamine for an efficient segregation of HOMO and LUMO densities. Such separation is ideal for the ICT transition and is attributed to the presence of a strong conjugated unit, of which thiophene is an example, in the given D–A–D system. Experimental estimation of the HOMO energies was carried out using photo electron spectroscopy in air (PESA) and the LUMO energies were calculated by adding the optical band gap (film spectra) to the HOMO values (see Figure 6 for energy level diagram and Figure S1 [see Supplementary Material] for the PESA curve). Film spectra indicated that insertion of thiophene reduces the band gap of **AS2** by 0.24 eV when compared with **AS1**. Furthermore, the estimation of HOMO using PESA revealed that the HOMO energy level of **AS2** was raised by 0.18 eV when compared with the HOMO level of **AS1**. This is in agreement with the DFT calculations that the presence of thiophene indeed plays a crucial role for: (1) density segregation; (2) tuning the optical energy levels; and (3) theoretical and experimental band gap reduction. These measurements and calculations provide a strong rational for our design strategy that the induction of a conjugated functionality can indeed improve the optoelectronic properties of a given chromophore. The energy level diagram advised that the band gaps of these materials are all in the range required of donor materials for BHJ devices. **AS2** optical band gap is somewhat narrower in magnitude than 2.0 eV measured for the conventional P3HT. The optical and electrochemical properties of both the materials in solution and film form are summarized in Supplementary Material Table S1.

Encouraging though the optoelectronic properties are, the compounds must display thermal stability given the rigorous conditions used in device fabrication such as annealing at temperature in the excess of 100 °C. In line with this requirement and to determine the thermal stability of **AS2** and **AS1**, thermogravimetric analysis (TGA) was conducted. TGA (Supplementary Material Figure S2) indicated that both **AS2** and **AS1** are thermally stable and will not degrade during the annealing of BHJ devices.

**Figure 7:**
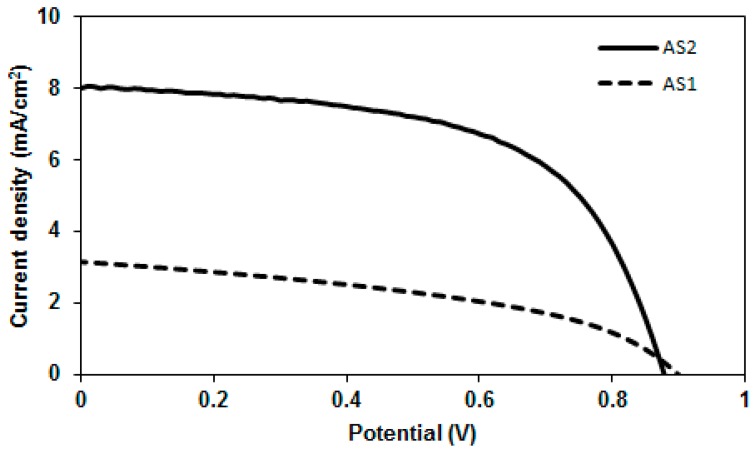
Current–voltage curves for the optimized devices based on AS2 and AS1 in blends with PC_61_BM (1:1 *w*/*w*) under simulated sunlight (AM 1.5, 1000 W·m^−2^). Device structure was: ITO/PEDOT-PSS (38 nm)/active layer/Ca (20 nm)/Al (100 nm) where the active layers were the blends of either **AS2** or **AS1** and PC_61_BM spun on top of the films of PEDOT:PSS using chlorobenzene solvent.

After correlating the optoelectronic properties of **AS2** and **AS1** with those of the conventional PC_61_BM acceptor (see Figure 6), we screened their potency as donor materials (p-type) in solution-processable BHJ devices under simulated sunlight and monochromatic light illumination. The blend solutions of both the materials and PC_61_BM were used to cast an active layer on top of the PEDOT:PSS surface. The BHJ device architecture used was ITO/PEDOT:PSS (38 nm)/active layer/Ca (20 nm)/Al (100 nm) where the active layer was a solution processed blend of either **AS2** or **AS1** and PC_61_BM. For **AS2**, a promising PCE of 4.10% was achieved when the film was spin-coated from a chlorobenzene solution as a 1:1 blend with PC_61_BM. By contrast, the maximum PCE obtained for a device based on **AS1** was 1.23%, when fabricated under similar conditions. The comparative current–voltage curves for the optimized blends of **AS2** and **AS1** with PC_61_BM are shown in Figure 7. High boiling solvents such as chlorobenzene are better not only from processing point of view but also for achieving smoother films without crystallization occurring on the active surfaces. Latter was particularly true as our efforts to construct BHJ devices using a low-boiling solvent, such as chloroform, afforded either uneven surfaces or minor cracks on the active surfaces.

The optimized devices based on **AS2** exhibited a decreased open circuit voltage (*V*_oc_) than the devices based on **AS1**. This in fact is consistent with the measured HOMO values where a higher HOMO for **AS2** would predict a lower *V*_oc_. On the other hand, the short circuit current density (*J*_sc_) for the devices fabricated using **AS2** was higher than the *J*_sc_ extracted from the devices based on **AS1**. This was in agreement with the observed bathochromic shift in the absorption spectrum of **AS2** compared with **AS1**. The photovoltaic cell parameters for **AS2**-based devices were 0.88 V [open circuit voltage, *V*_oc_], 8.01 mA·cm^−2^ [current density, (*J*_sc_)], 0.58 [fill factor, (FF)] and 4.10% [power conversion efficiency, (PCE)]. Initial screening of the BHJ devices based on **AS1**:PC_61_BM showed moderate device performance with a high *V*_oc_ of 0.90 V, FF of 43% *J*_sc_ of 3.15 mA/cm^2^ and an overall PCE of 1.23%. Taken as a whole, the insertion of thiophene functionality into the studied D–A–D structural motif incorporating PDA acceptor functionality resulted in significant enhancement of *J*_sc_ and PCE values by factors >2 and >3, respectively, thus promoting the use of a smaller conjugated functionality, such as thiophene, as an interesting structural concept for the design and development of highly efficient BHJ materials. Furthermore, it is notable to mention that **AS2** showed optimal performance with inexpensive PC_61_BM and simple device architecture, thus providing some strong incentive to apply the design concept reported in this work to the new generations of BHJ materials.

**Figure 8:**
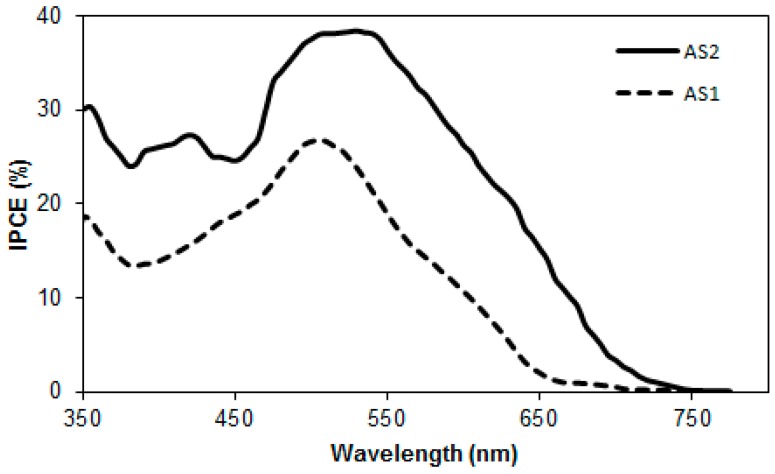
IPCE spectra of **AS2** and **AS1** with PC_61_BMblends.

The incident photon-to-current-conversion efficiency (IPCE) spectra of these BHJ devices are shown in Figure 8. The IPCE measurement of these BHJ devices was broad spectrum, typically covering most of the visible range, from 350 to 750 nm. **AS2** and **AS1** exhibited high plateaus at ~40% and ~27% for the best BHJ devices respectively. The IPCE spectrum of **AS2**, which carried a thiophene functionality, was red-shifted when compared with **AS1**, a finding that is consistent with the result of thin film absorption spectrum. The significantly higher peak IPCE of **AS2** compared to **AS1** indicated that the superior performance of **AS2** can be rationalized in terms of enhanced light-harvesting and appropriately tuned optical energy levels, thereby corroborating the design principle.

**Figure 9:**
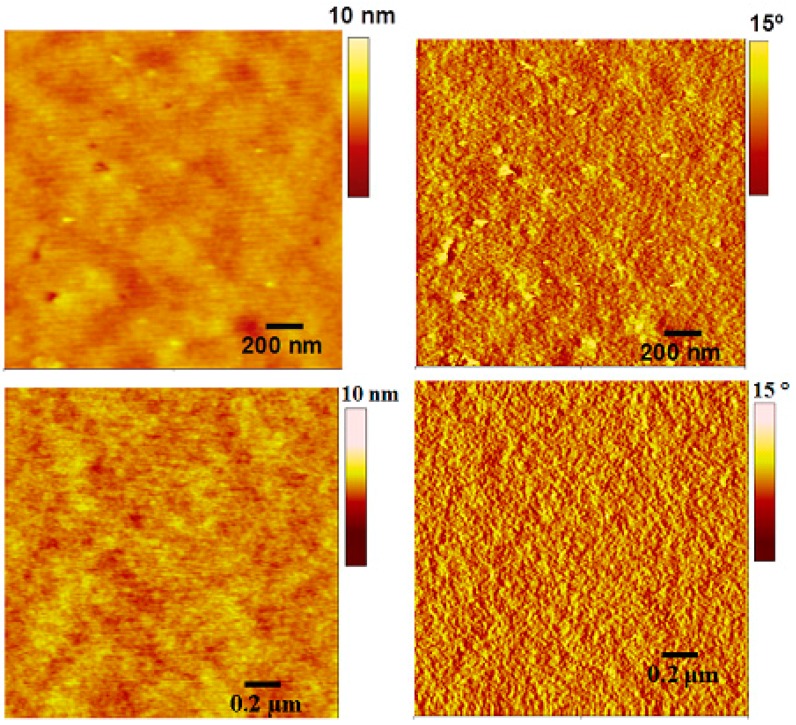
AFM images of 1:1 blends of **AS2** (**top**) and **AS1** (**bottom**) with PC_61_BM as-casted from chlorobenzene solution. Topographic (**left**) and phase images (**right**) are shown.

To examine the physical microstructure of the blend surface, we used atomic force microscopy (AFM) in tapping mode. The actual surface morphology of the blend films of **AS2**/**AS1**:PC_61_BM (1:1 *w*/*w*) is shown in Figure 9. Physically, both the blends were found to be smooth and the root mean square roughness of 0.41 nm and 0.35 nm was observed for **AS2** and **AS1** respectively. No cracks were observed on the film surfaces when the films were spin-casted using chlorobenzene (3000 rpm). The processing of active films of BHJ devices using a high boiling solvent such as chlorobenzene is advantageous over low boiling solvent such as chloroform and is in agreement with the AFM morphology. Our attempts to fabricate BHJ devices using chloroform resulted in very poor photovoltaic performance. This was mainly due to inferior film quality. Though **AS2** exerted promising PCE in this preliminary work, ample scope still exists to explore device strategies to enhance PCE. The performance might be improved by (1) using PC_71_BM or (2) effective interlayer, such as metal oxide interlayer, which can facilitate the efficient charge extraction, and (3) devising processing methods, such as use of additives. Work towards some of such strategies is the subject of on-going work in our laboratories. The discovery of potential materials, such as **AS2**, exhibiting promising optoelectronic and photovoltaic properties opens up the way to develop D–A–D modular small organic chromophores, with the use of PDA acceptor in particular, and paves the way for such materials to be used for other organic electronic applications.

## 3. Experimental Section

### 3.1. Materials and Instruments

All reagents and chemicals used, unless otherwise specified, were purchased from Sigma-Aldrich (Sydney, Australia). The solvents used for reactions were obtained from Merck Speciality Chemicals (Sydney, Australia) and were used as received. Unless otherwise specified, all ^1^H- and ^13^C-NMR spectra were recorded using a Bruker AV300 spectrometer at 300 and 75 MHz or a Bruker AV400 spectrometer at 400 and 100 MHz, respectively (Bruker Corporation, Billerica, MA, USA). Chemical shifts (δ) are reported in parts per million (ppm). Thin-layer chromatography (TLC) was performed using 0.25 mm thick plates precoated with Kieselgel 60 F_254_ silica gel (Merck, Darmstadt, Germany) and visualized using UV light (254 nm and 365 nm). Melting points were measured using a MPD350 digital melting point apparatus (Gallenkamp, Sanyo, Osaka, Japan) and are uncorrected. High-resolution mass spectra experiments were carried out on a Q-Exactive FTMS (Thermo Scientific, Bremen, Germany) ionizing by atmospheric-pressure chemical ionization (APCI) from an ASAP probe. All UV-Vis absorption spectra were recorded on a Hewlett Packard (HP) 8453 diode array UV-Vis spectrophotometer (Agilent Technologies, Mulgrave Victoria, Australia). Thin films were spin-coated from chlorobenzene at a spin speed of 2000 rpm for 1 min onto cleaned glass slides. PESA measurement was recorded using a Riken Keiki AC-2 PESA spectrometer (RKI Instruments, Union City, CA, USA) with a power setting of 5 nW and a power number of 0.5. Samples for PESA were prepared on clean glass substrates. Fabrication and characterization of BHJ devices, and preparation of thin-film transistors has been reported previously [18,30].

### 3.2. Synthesis and Characterization of Target Molecules

#### 3.2.1. Synthesis of **AS1**

1,4-Phenylenediacetonitrile (500 mg, 3.21 mmol) was added to the mixture of 4-(diphenyl-amino)benzaldehyde (1.84 g, 6.74 mmol) in methanol (50.0 mL) at room temperature and the resulting mixture was heated at reflux overnight. The precipitated solid was collected by filtration, washed with methanol and dried under vacuum to give 1.85 g (86.3%) of **AS1** as an orange powder. m. p. 242–245 °C; HPLC (5% H_2_O/ACN): 97.6%; IR (solid film, cm^−1^) 3061, 3035 (Ar -CH str), 2250, 2206 (-CN str), 1580, 1504, 1486 (Ar C=C str), 1330, 1285, 1192, 1179; ^1^H-NMR (400 MHz, CDCl_3_): δ = 7.80–7.76 (m, 4 H), 7.68 (s, 4 H), 7.45 (s, 2 H), 7.33–7.28 (m, 8 H), 7.16–7.10 (m, 12 H), 7.05–7.02 (m, 4 H); ^13^C-NMR (400 MHz, CDCl_3_): δ = 150.2, 146.5, 141.8, 135.1, 130.8, 129.6, 126.2, 126.1, 125.8, 124.5, 120.7, 118.5, 106.6; HRMS (APCI): calculated for C_48_H_35_N_4_ [M+H]^+^ 667.2856; found 667.2851.

#### 3.2.2. Synthesis of 5-(4-(Diphenylamino)phenyl)thiophene-2-carbaldehyde (**1**)

A solution of 1,2-dimethoxyethane (DME, 40.0 mL) and 2M Na_2_CO_3_ (20.0 mL) is degassed with nitrogen (N_2_) for 30 min. To this solution 5-bromothiophene-2-carbaldehyde (191 mg, 1.00 mmol) and (4-(diphenylamino)phenyl)boronic acid (433 mg, 1.50 mmol) were added and the mixture was heated at 60 °C for 30 min. [Pd(PPh_3_)_4_] (110 mg, 0.10 mmol) was added and the resulting mixture was stirred at 90 °C overnight. The reaction mixture was extracted with diethyl ether (3 × 50 mL). The organic layers were combined, washed with brine (100 mL) and dried over anhydrous magnesium sulfate. The solvent was evaporated to afford crude yellow solid, which was crystallized from hexane and chloroform to yield 378 mg (71%) of **1** as yellow needle like crystals. m. p. 100–102 °C; IR (solid film, cm^−1^): 3313, 3023, 2794, 1956, 1887, 1720, 1659, 1584, 1527, 1485, 1465, 1324, 1261, 1226, 1177, 1153, 1075, 1054; ^1^H-NMR (300 MHz, CD_2_Cl_2_): δ = 9.87 (s, 1H), 7.75 (d, *J* = 6.8 Hz, 1H), 7.60–7.57 (m, 2H), 7.37–7.31 (m, 5H), 7.71–7.15 (m, 6H), 7.13–7.06 (m, 2H); ^13^C-NMR (300 MHz, CDCl_3_): δ = 182.9, 154.5, 149.5, 147.4, 141.8, 138.1, 129.8, 127.6, 126.5, 125.6, 124.3, 123.4, 122.6; LRMS (ESI; 2% Formic acid): *m*/*z* = 356 (M + H)^+^; HRMS (APCI): calculated for C_23_H_17_NOS [M]^+^ 355.1031; found 355.1026.

#### 3.2.3. Synthesis of **AS2**

A solution of sodium ehtoxide was prepared by dissolving one pellet (approx. 200 mg) of sodium hydroxide (NaOH) in ethanol (EtOH, 20 mL) with mild heating (40 °C). To this solution PDA (78 mg, 0.50 mmol) was added and allowed to dissolve. A solution of **1** (390 mg, 1.1 mmol) in EtOH (10 mL) was added dropwise and the resulting solution was refluxed for 6 h. The mixture was allowed to cool to room temperature and placed in an ice-bath for 30 min. The separated red solid was filtered off, washed with cold EtOH (50.0 mL) followed by cold hexane (50.0 mL), and dried under high vacuum at 40 °C. The solid was crystallized using CHCl_3_/hexane to afford 574 mg of **AS2** (63%) as a brick-red solid. m. p. >240 °C (decomposed at 240 °C); IR (solid film, cm^−1^) 3674, 2988, 2208 (-CN str), 1578, 1486, 1440, 1421, 1324, 1271, 1238, 1179, 1064, 922, 829. ^1^H-NMR (300 MHz, CD_2_Cl_2_): δ = 7.77–7.76 (m, 3H), 7.65–7.63 (m, 1H), 7.60–7.59 (m, 2H), 7.36–7.31 (m, 5H) 7.18–7.15 (m, 5H), 7.12–7.08 (m, 3H); ^13^C-NMR (300 MHz, CDCl_3_) δ = 149.9, 148.6, 147.1, 136.1, 134.8, 134.7, 134.4, 129.4, 127.0, 126.7, 126.0, 124.9, 123.6, 122.8, 122.7, 118.1, 105.7; LRMS (MALDI-TOF): *m*/*z* = 830.229; HRMS (APCI): calculated for C_56_H_39_N_4_S_2_ [M + H]^+^ 831.2611; found 831.2602.

#### 3.2.4. X-ray Crystallography

CCDC’s **1420377** for **1** and **1420378** for **AS2** contain the supplementary crystallographic data for this paper. These data can be obtained free of charge via http://www.ccdc.cam.ac.uk/conts/retrieving.html (or from the CCDC, 12 Union Road, Cambridge CB2 1EZ, UK; Fax: +44 1223 336033; E-mail: deposit@ccdc.cam.ac.uk).

## 4. Conclusions

In conclusion, we have demonstrated the first use of the PDA acceptor functionality in conjunction with a thiophene unit for the design and development of a BHJ chromophore, **AS2**, where PDA was used in the D–A–D modular arrangement. The incorporation of this strong conjugating thiophene unit helped to improve light-harvesting, photocurrent density and PCE of **AS2** when compared with an analogue, **AS1**. The incorporation of the thiophene functionality was of clear benefit in improving the BHJ performance and indicates a potential to be broadly applicable in the design and development of future high performance BHJ chromophores.

## Supplementary Materials

Supplementary materials can be accessed at: http://www.mdpi.com/1420-3049/20/12/19798/s1.
