# Supplementary material for: Significant Improvement of Optoelectronic and Photovoltaic Properties by Incorporating Thiophene in a Solution-Processable D–A–D Modular Chromophore
Source: Molecules. 2015 Dec 4;20(12):21787–801. doi: 10.3390/molecules201219798 (PMC6332373; doi:10.3390/molecules201219798)
Supplement: Supplementary file 1 [file molecules-20-19798-s001.pdf]

Supplementary Materials: Significant Improvement of Optoelectronic and Photovoltaic Properties by Incorporating Thiophene in a Solution-Processable D–A–D Modular Chromophore

Aaron M. Raynor, Akhil Gupta, Christopher M. Plummer, Sam L. Jackson, Ante Bilic, Hemlata Patil, Prashant Sonar and Sheshanath V. Bhosale

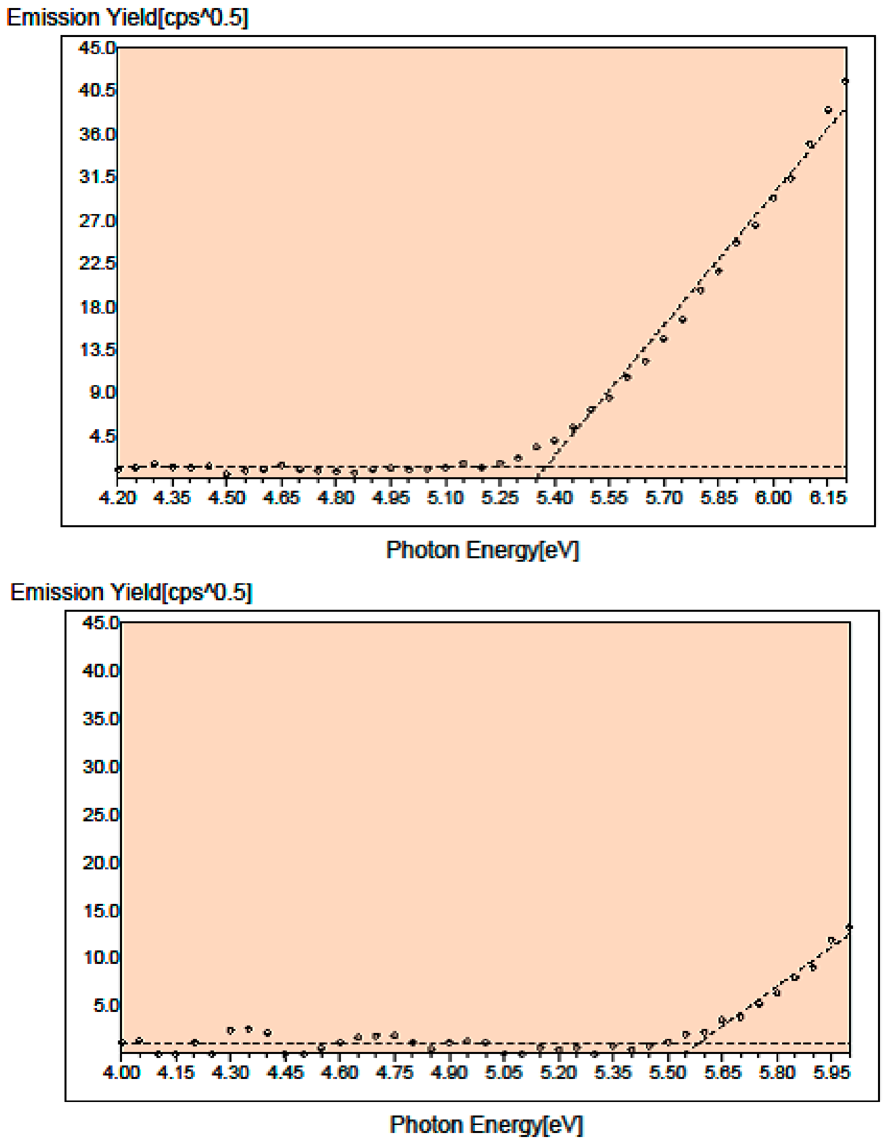

Figure S1. PESA spectra of thin films of **AS2** (upper) and **AS1** (lower). The dashed-lines show the fits to extract ionisation potentials which correspond to the HOMO energy levels.

Table S1. Comparative optoelectronic properties of **AS2** and **AS1**

Material	Absorption (Solution) λ_{abs} ᵃ /(nm)	Extinction Coefficient (ε/($M^{-1}\cdot cm^{-1}$))	Absorption (Film) λ_{abs} ᵇ/onset /(nm)	Energy Levels ᶜ (HOMOs/LUMOs) /(eV)		$E_{bandgap}$/eV ᵈ	
				Theoretical	Experimental	Theoretical	Experimental
**AS2**	459	59,057	497/640	5.28/2.83	5.38/3.45	2.45	1.93
**AS1**	436	49,125	470/570	5.34/2.55	5.56/3.39	2.79	2.17

[a] UV–Vis absorption in chloroform solution; [b] Longest wavelength of UV–Vis absorption maxima for as-casted films from chloroform solutions; [c] Theoretical energy levels were calculated using the Gaussian 09 suite of programs and the B3LYP/6-311+G(d,p)//B3LYP/6-31G(d) level of theory. Experimental energy levels were measured/calculated using a combination of PESA and the optical absorption; [d] Optical band gaps were calculated/measured under similar conditions reported for c.

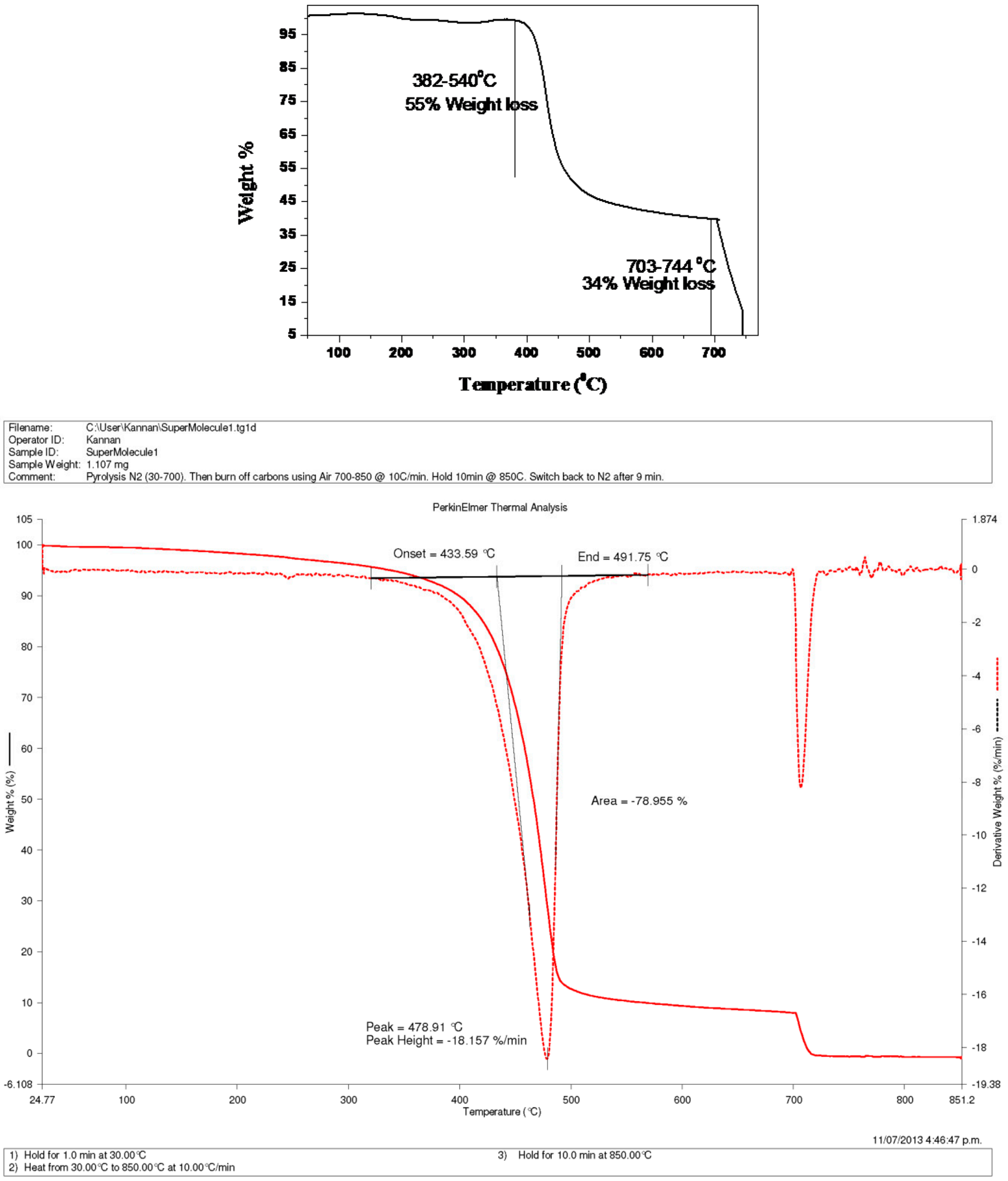

Figure S2. TGA curves of **AS2** (upper) and **AS1** (lower).

Spectra of AS1

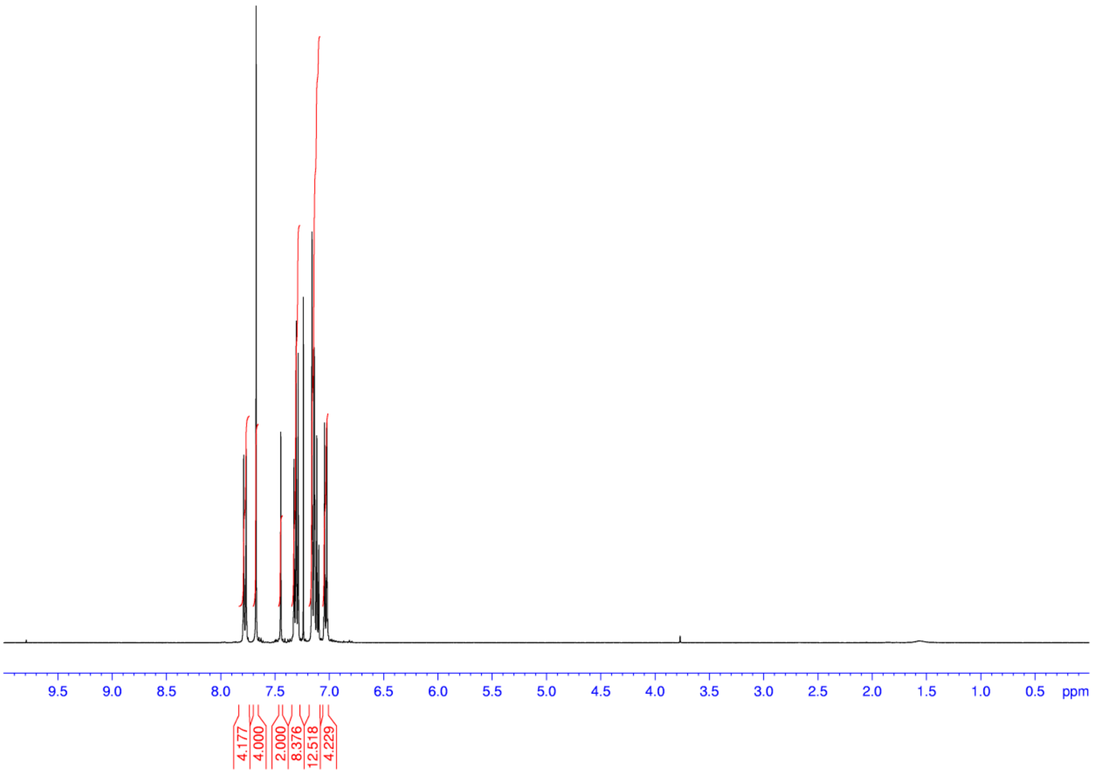

Figure S3. ^{1}H-NMR spectrum in $CDCl_3$.

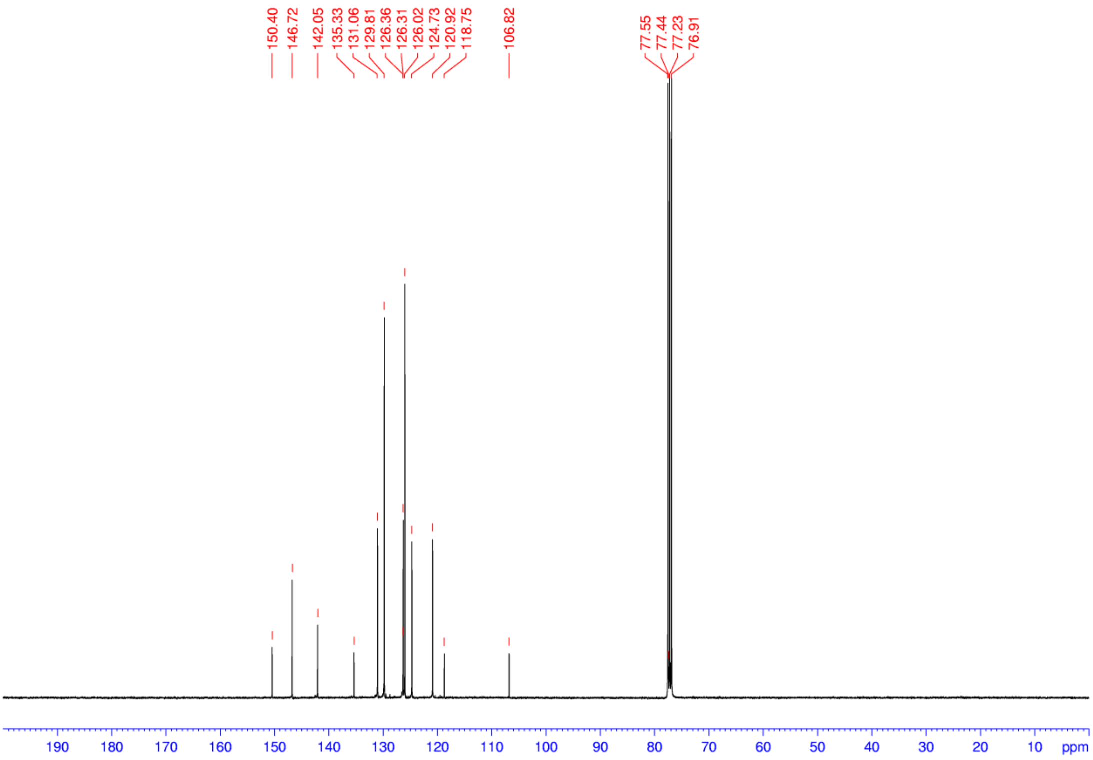

Figure S4. ^{13}C-NMR spectrum in $CDCl_3$.

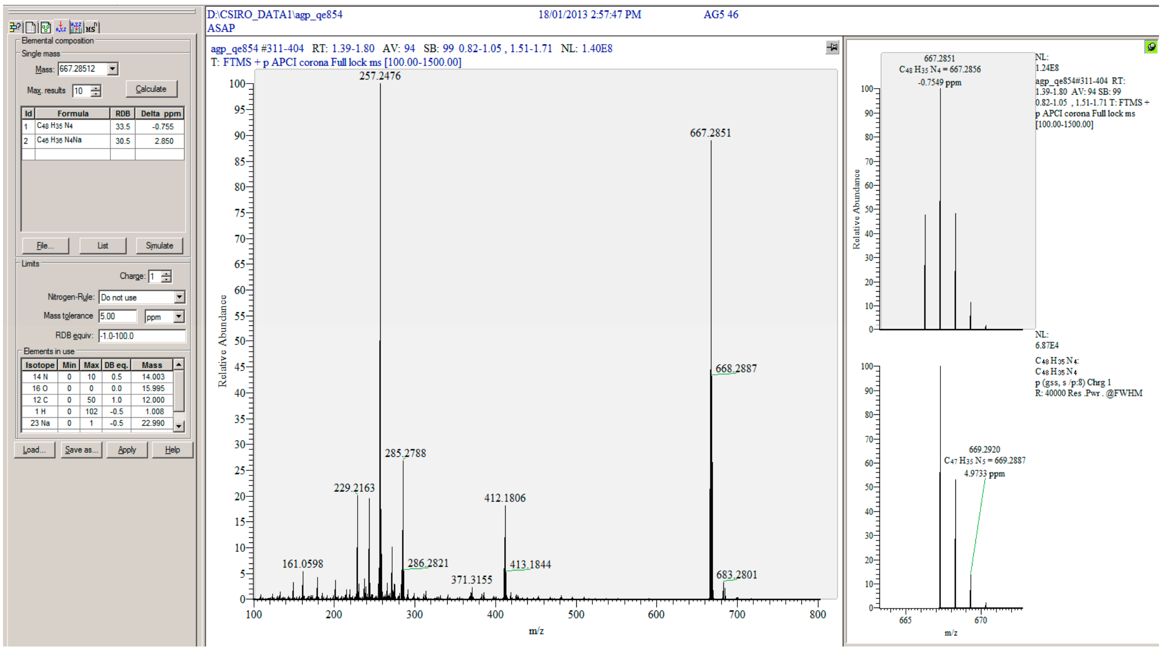

Figure S5. HRMS spectrum.

Spectra of Intermediate 1

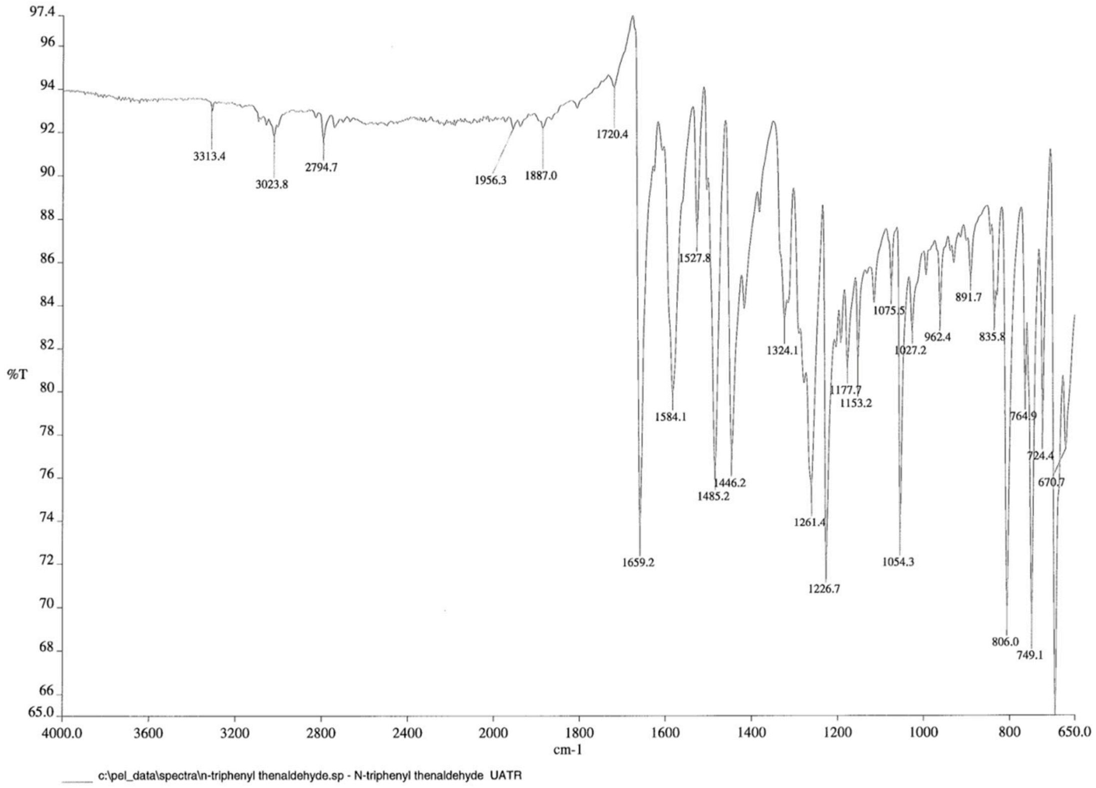

Figure S6. IR spectrum.

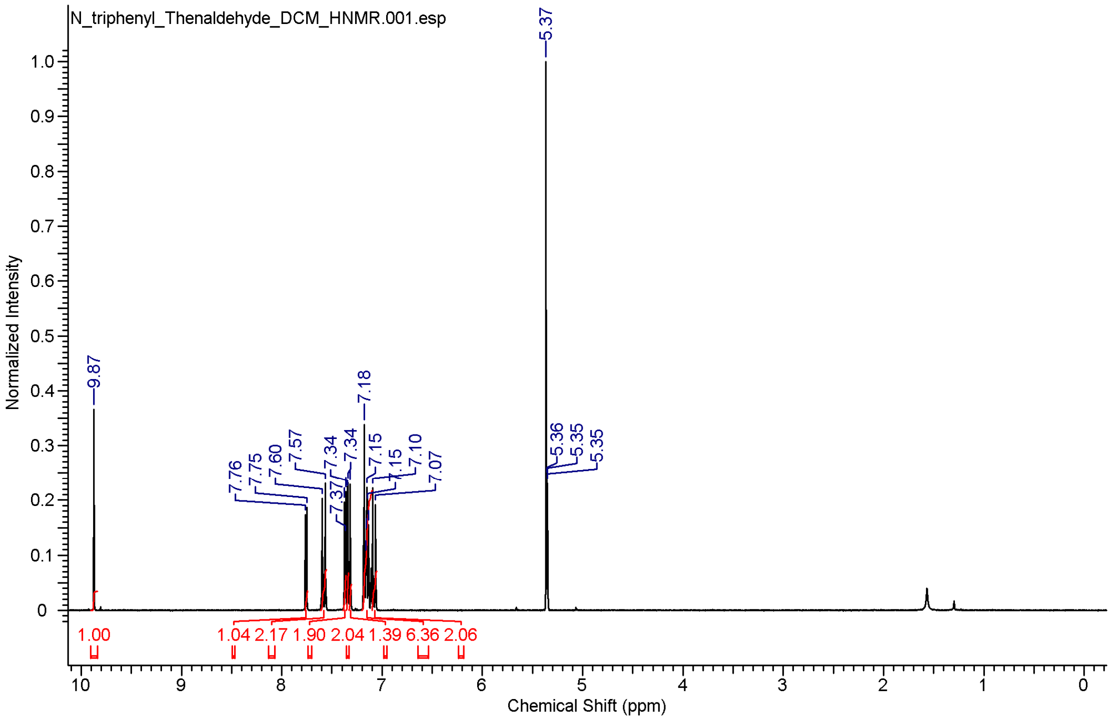

Figure S7. ^{1}H-NMR spectrum in CD_2Cl_2.

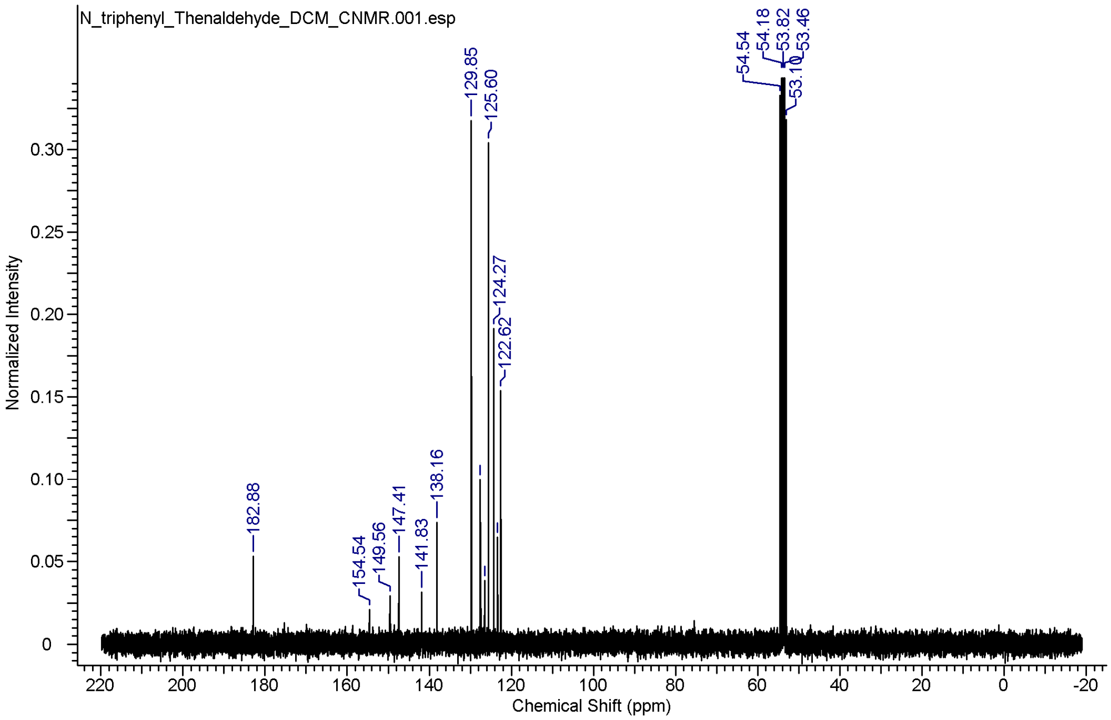

Figure S8. ^{13}C-NMR spectrum in CD_2Cl_2.

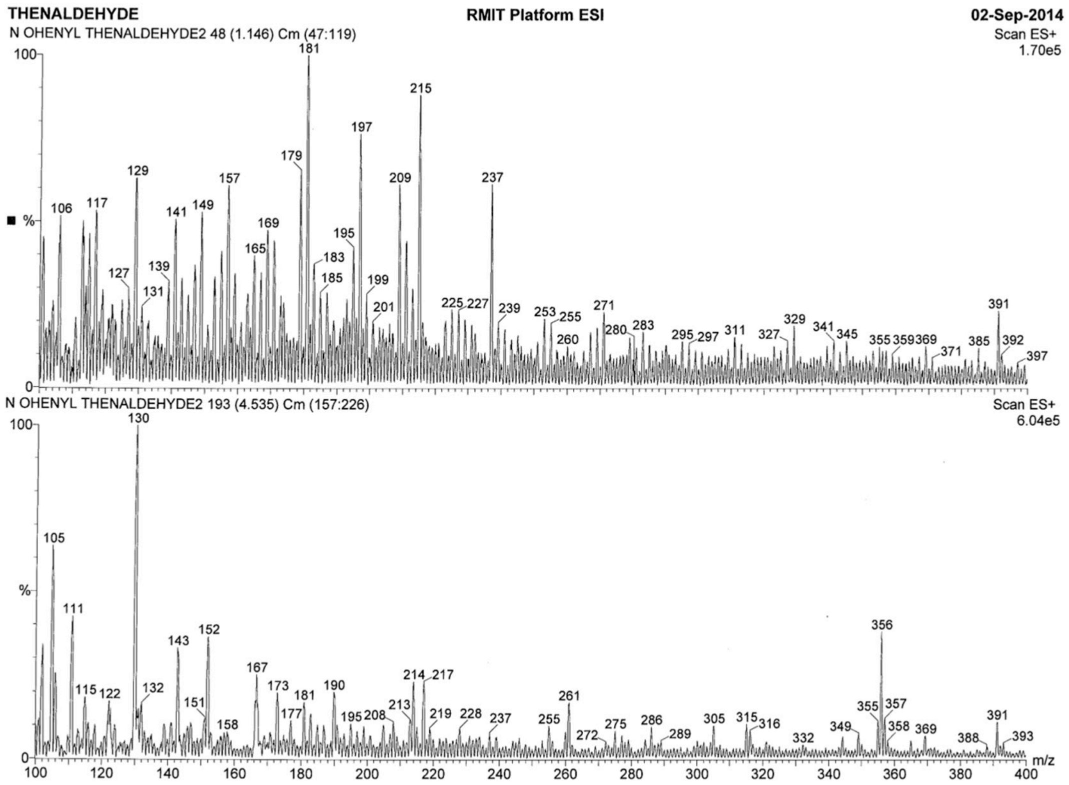

Figure S9. LRMS spectrum.

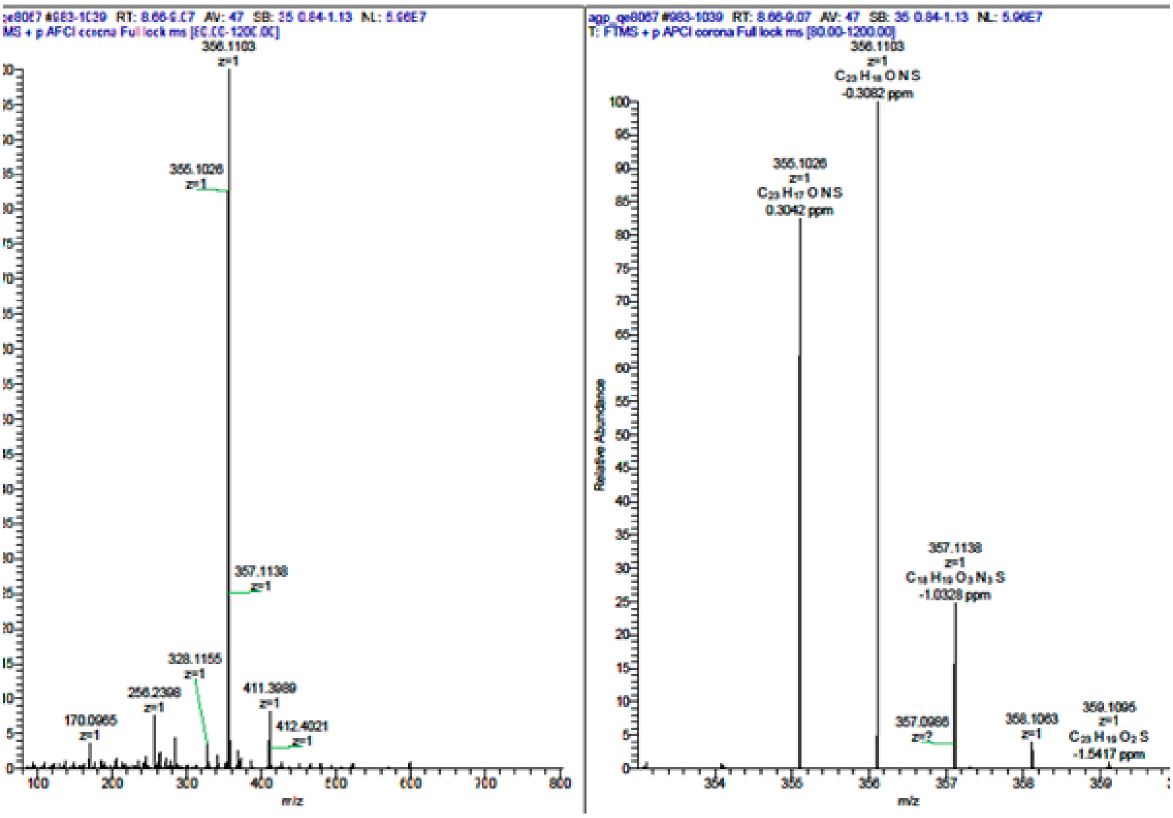

Figure S10. HRMS spectrum.

Spectra of AS2

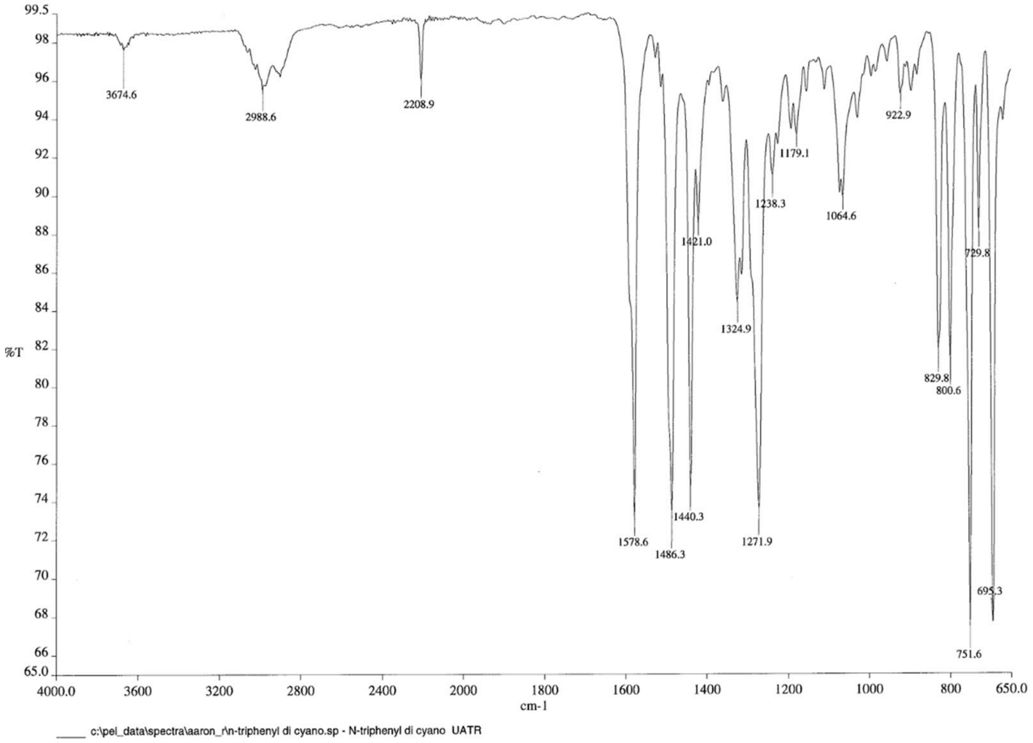

Figure S11. IR spectrum.

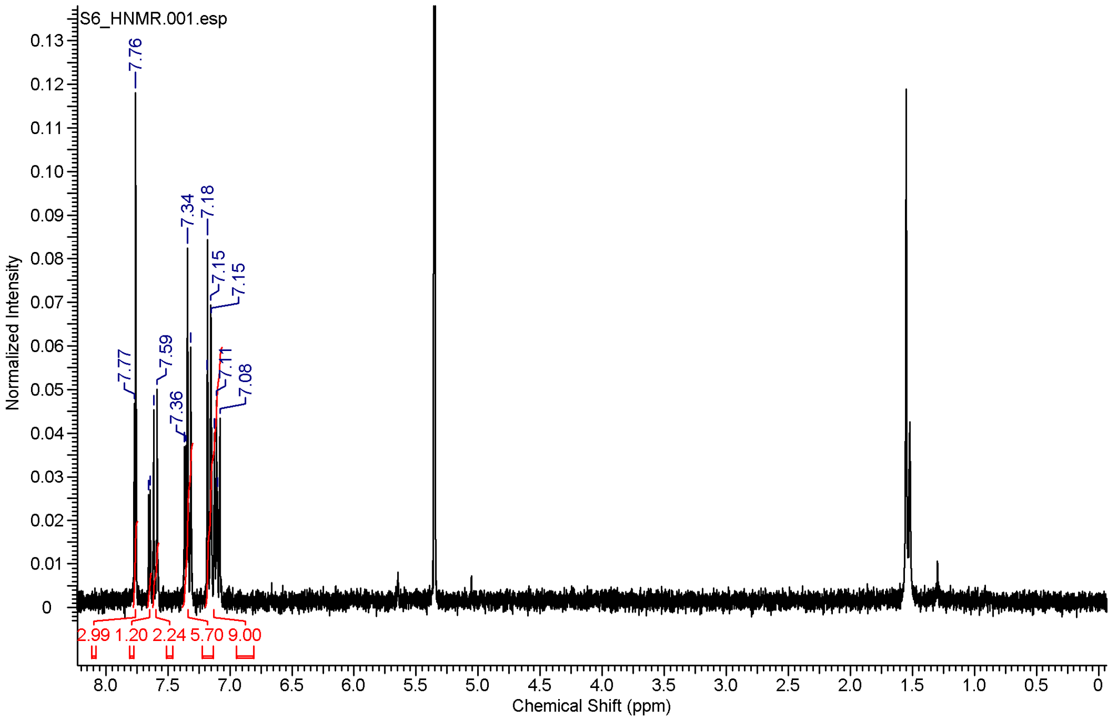

Figure S12. ^{1}H-NMR spectrum in CD_2Cl_2.

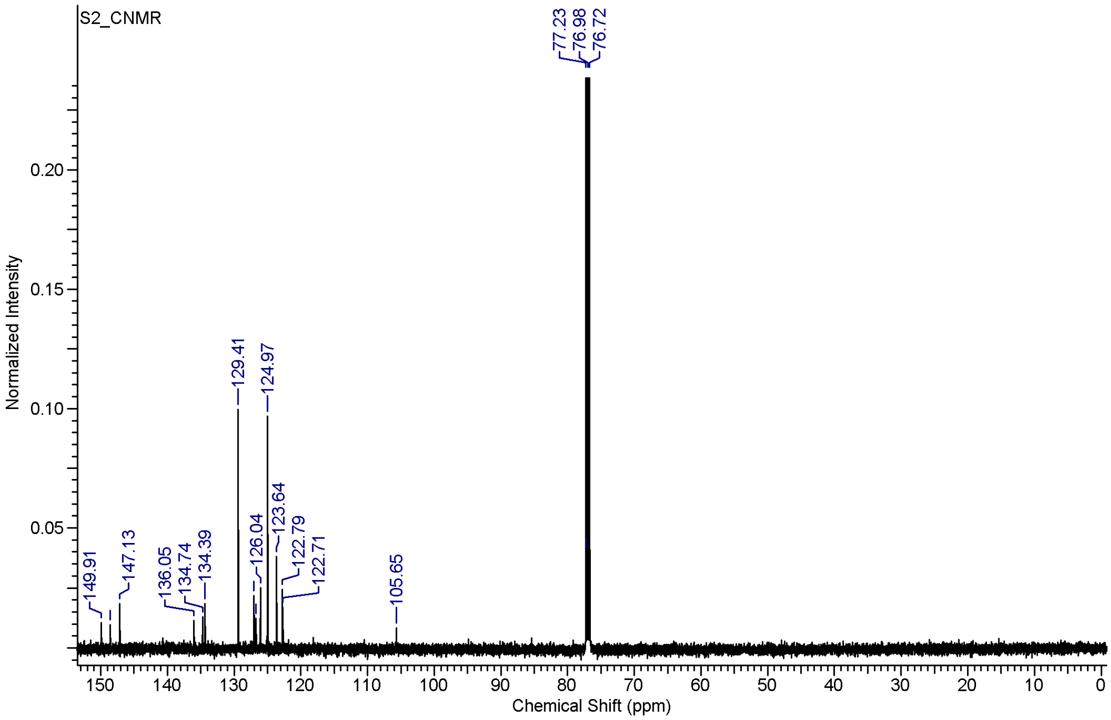

Figure S13. ^{13}C-NMR spectrum in $CDCl_3$.

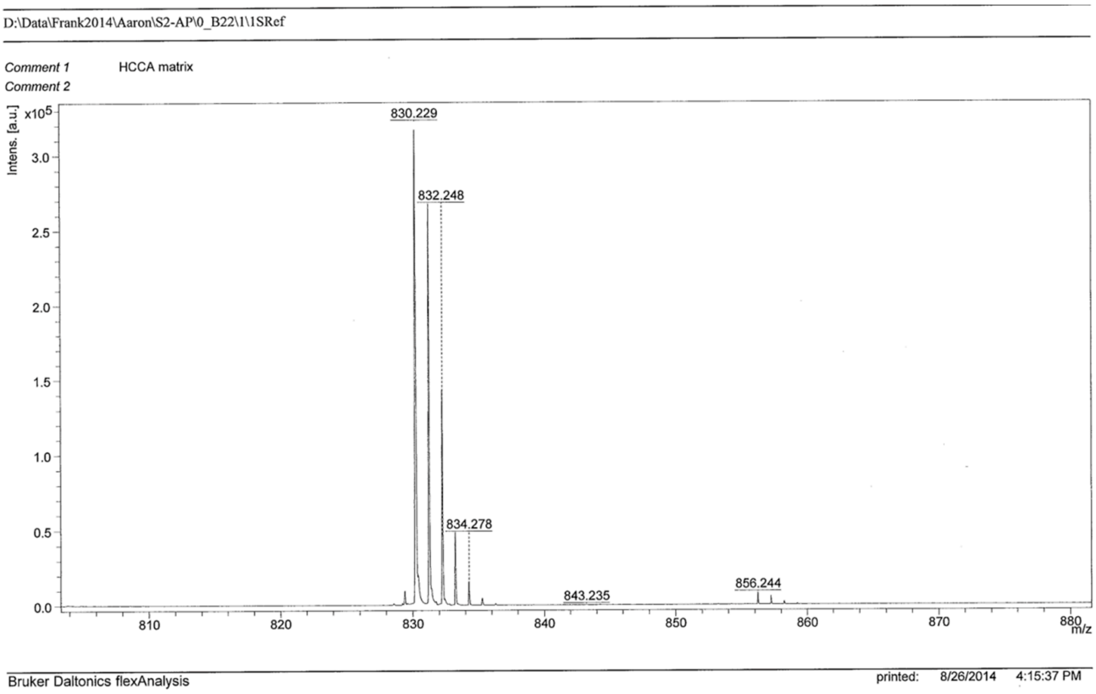

Figure S14. LRMS (MALDI-TOF) spectrum.

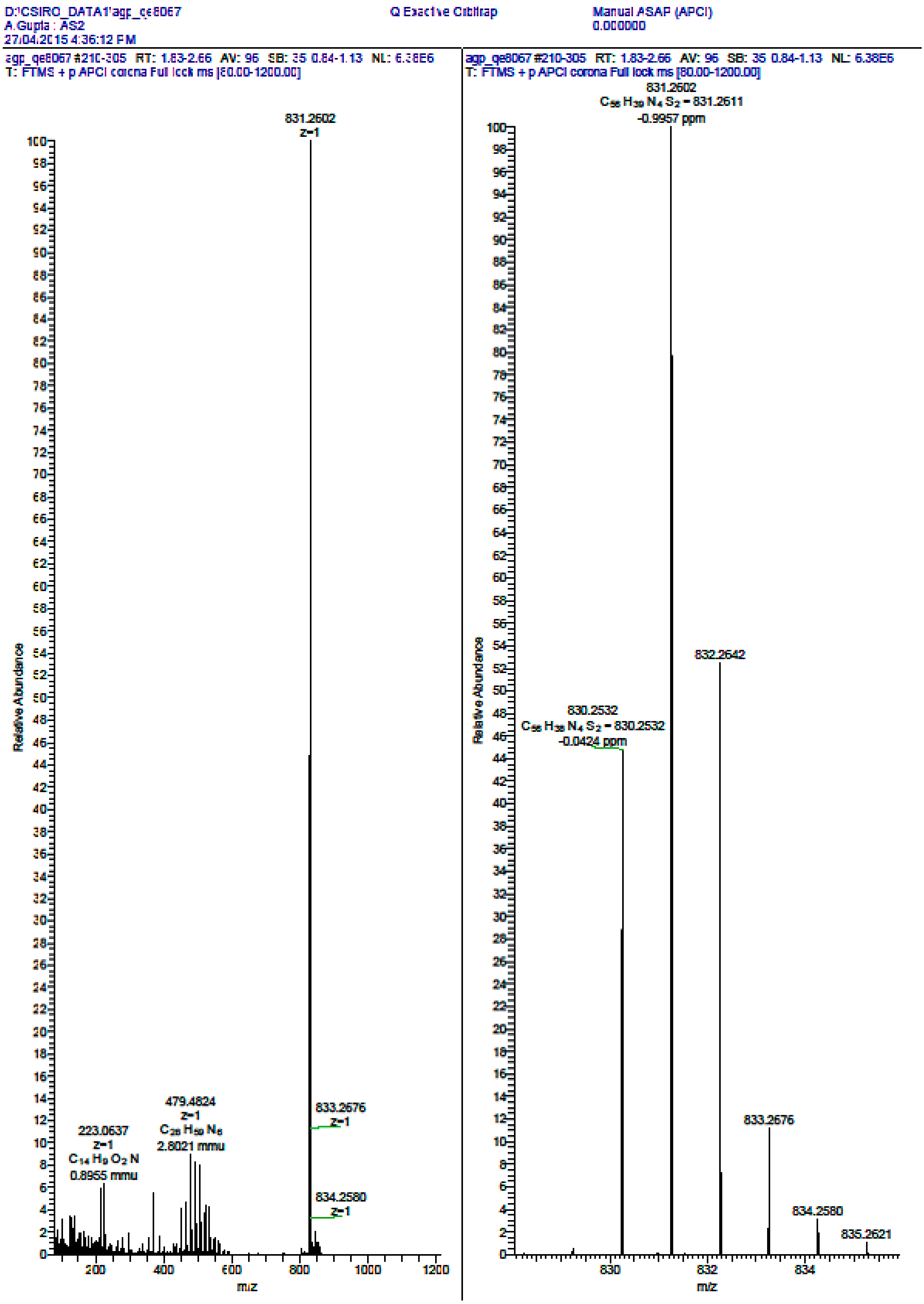

Figure S15. HRMS spectrum.

Check CIF of AS2

checkCIF/PLATON report

Structure factors have been supplied for datablock(s) shelxl

THIS REPORT IS FOR GUIDANCE ONLY. IF USED AS PART OF A REVIEW PROCEDURE FOR PUBLICATION, IT SHOULD NOT REPLACE THE EXPERTISE OF AN EXPERIENCED CRYSTALLOGRAPHIC REFEREE.

No syntax errors found. CIF dictionary Interpreting this report

Datablock: shelxl

```
Bond precision:   C-C = 0.0038 A          Wavelength=0.71073

Cell:             a=7.1570(19)      b=12.326(3)      c=14.492(4)
                  alpha=82.712(6)   beta=89.825(7)   gamma=86.756(6)
Temperature:      200 K

                   Calculated                    Reported
Volume             1266.1(6)                     1266.1(6)
Space group        P -1                          P -1
Hall group         -P 1                          -P 1
Moiety formula     C56 H38 N4 S2, 2(C H Cl3)     C56 H38 N4 S2, 2(C H Cl3)
Sum formula        C58 H40 Cl6 N4 S2             C58 H40 Cl6 N4 S2
Mr                 1069.76                       1069.76
Dx,g cm-3          1.403                         1.403
Z                  1                             1
Mu (mm-1)          0.466                         0.464
F000               550.0                         550.0
F000'              551.29
h,k,lmax           10,18,22                      10,18,22
Nref               9484                          9484
Tmin,Tmax          0.889,0.984                   0.889,0.984
Tmin'              0.873

Correction method= # Reported T Limits: Tmin=0.889 Tmax=0.984
AbsCorr = MULTI-SCAN

Data completeness= 1.000           Theta(max)= 32.950

R(reflections)= 0.0682( 5479)      wR2(reflections)= 0.2391( 9484)

S = 1.017                  Npar= 316
```

The following ALERTS were generated. Each ALERT has the format
test-name_ALERT_alert-type_alert-level.
Click on the hyperlinks for more details of the test.

Figure S16. *Cont.*

```
Alert level C
PLAT244_ALERT_4_C Low   'Solvent' Ueq as Compared to Neighbors of        C29 Check
PLAT790_ALERT_4_C Centre of Gravity not Within Unit Cell: Resd.  #         1 Note
              C56 H38 N4 S2
```

```
Alert level G
PLAT066_ALERT_1_G Predicted and Reported Tmin&Tmax Range Identical         ? Check
PLAT072_ALERT_2_G SHELXL First  Parameter in WGHT Unusually Large.      0.12 Report
PLAT804_ALERT_5_G Number of ARU-Code Packing Problem(s) in PLATON          1 Info
```

```
  0 ALERT level A = Most likely a serious problem - resolve or explain
  0 ALERT level B = A potentially serious problem, consider carefully
  2 ALERT level C = Check. Ensure it is not caused by an omission or oversight
  3 ALERT level G = General information/check it is not something unexpected

  1 ALERT type 1 CIF construction/syntax error, inconsistent or missing data
  1 ALERT type 2 Indicator that the structure model may be wrong or deficient
  0 ALERT type 3 Indicator that the structure quality may be low
  2 ALERT type 4 Improvement, methodology, query or suggestion
  1 ALERT type 5 Informative message, check
```

It is advisable to attempt to resolve as many as possible of the alerts in all categories. Often the minor alerts point to easily fixed oversights, errors and omissions in your CIF or refinement strategy, so attention to these fine details can be worthwhile. In order to resolve some of the more serious problems it may be necessary to carry out additional measurements or structure refinements. However, the purpose of your study may justify the reported deviations and the more serious of these should normally be commented upon in the discussion or experimental section of a paper or in the "special_details" fields of the CIF. checkCIF was carefully designed to identify outliers and unusual parameters, but every test has its limitations and alerts that are not important in a particular case may appear. Conversely, the absence of alerts does not guarantee there are no aspects of the results needing attention. It is up to the individual to critically assess their own results and, if necessary, seek expert advice.

Publication of your CIF in IUCr journals

A basic structural check has been run on your CIF. These basic checks will be run on all CIFs submitted for publication in IUCr journals (*Acta Crystallographica*, *Journal of Applied Crystallography*, *Journal of Synchrotron Radiation*); however, if you intend to submit to *Acta Crystallographica Section C* or *E*, you should make sure that full publication checks are run on the final version of your CIF prior to submission.

Publication of your CIF in other journals

Please refer to the *Notes for Authors* of the relevant journal for any special instructions relating to CIF submission.

PLATON version of 29/01/2015; check.def file version of 29/01/2015

Figure S16. *Cont.*

Datablock shelxl - ellipsoid plot

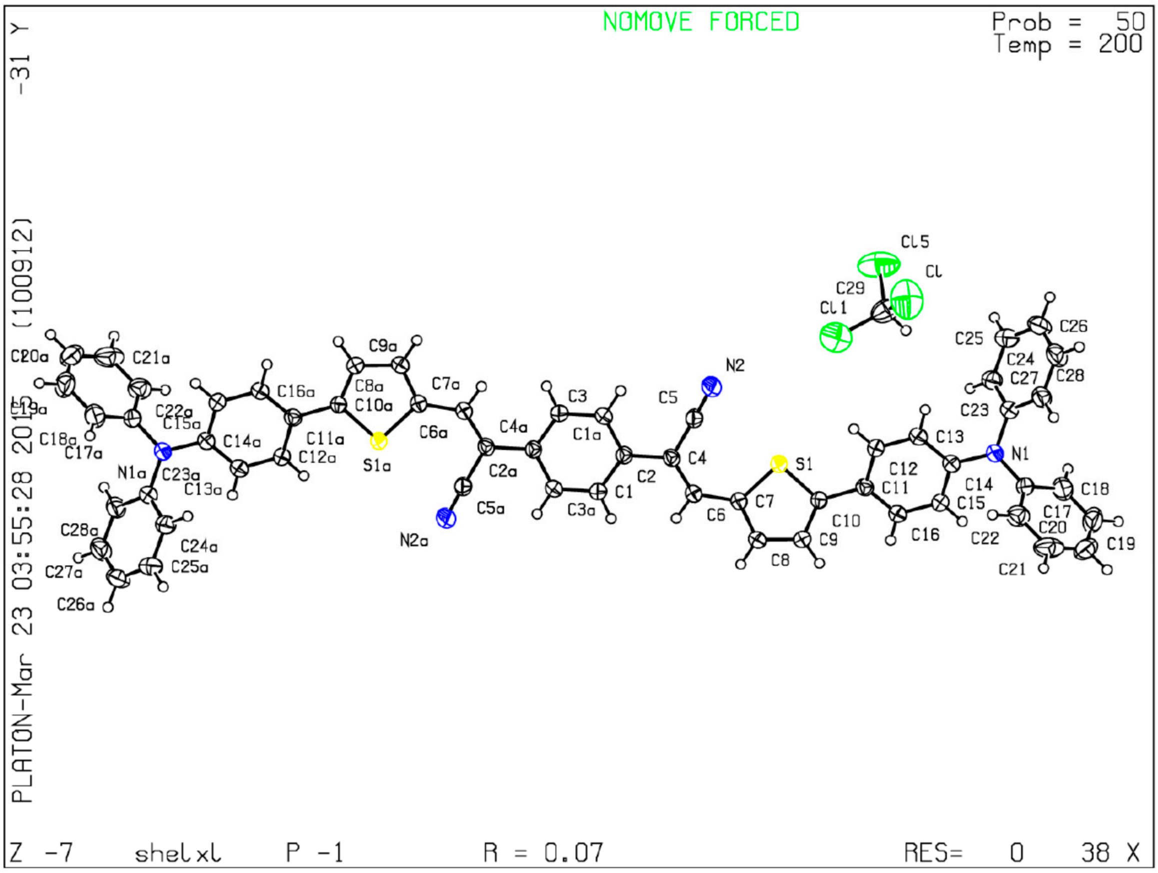

Figure S16. CIF of AS2.

Check CIF of aldehyde (1)

checkCIF/PLATON report

Structure factors have been supplied for datablock(s) shelxl

THIS REPORT IS FOR GUIDANCE ONLY. IF USED AS PART OF A REVIEW PROCEDURE FOR PUBLICATION, IT SHOULD NOT REPLACE THE EXPERTISE OF AN EXPERIENCED CRYSTALLOGRAPHIC REFEREE.

No syntax errors found. CIF dictionary Interpreting this report

Datablock: shelxl

```
Bond precision:   C-C = 0.0041 A          Wavelength=0.71073

Cell:             a=19.916(3)     b=6.6680(9)        c=13.4336(16)
                  alpha=90        beta=96.613(3)     gamma=90
Temperature:      200 K

                  Calculated                   Reported
Volume            1772.1(4)                    1772.1(4)
Space group       P 21/c                       P 21/c
Hall group        -P 2ybc                      -P 2ybc
Moiety formula    C23 H17 N O S                C23 H17 N O S
Sum formula       C23 H17 N O S                C23 H17 N O S
Mr                355.44                       355.43
Dx,g cm-3         1.332                        1.332
Z                 4                            4
Mu (mm-1)         0.194                        0.194
F000              744.0                        744.0
F000'             744.77
h,k,lmax          23,7,15                      23,7,15
Nref              2883                         2883
Tmin,Tmax         0.982,0.988                  0.982,0.988
Tmin'             0.945

Correction method= # Reported T Limits: Tmin=0.982 Tmax=0.988
AbsCorr = MULTI-SCAN

Data completeness= 1.000           Theta(max)= 24.294

R(reflections)= 0.0412( 2029)      wR2(reflections)= 0.1274( 2883)

S = 0.949                    Npar= 235
```

The following ALERTS were generated. Each ALERT has the format
test-name_ALERT_alert-type_alert-level.
Click on the hyperlinks for more details of the test.

Figure S17. *Cont.*

```
 Alert level C
CRYSC01_ALERT_1_C No recognised colour has been given for crystal colour.
THETM01_ALERT_3_C  The value of sine(theta_max)/wavelength is less than 0.590
            Calculated sin(theta_max)/wavelength =    0.5789
PLAT230_ALERT_2_C Hirshfeld Test Diff for    C15    --  C16      ..         6.0 su
PLAT340_ALERT_3_C Low Bond Precision on  C-C Bonds ...............      0.0041 Ang.
```

```
 Alert level G
PLAT066_ALERT_1_G Predicted and Reported Tmin&Tmax Range Identical          ? Check
```

```
   0 ALERT level A = Most likely a serious problem - resolve or explain
   0 ALERT level B = A potentially serious problem, consider carefully
   4 ALERT level C = Check. Ensure it is not caused by an omission or oversight
   1 ALERT level G = General information/check it is not something unexpected

   2 ALERT type 1 CIF construction/syntax error, inconsistent or missing data
   1 ALERT type 2 Indicator that the structure model may be wrong or deficient
   2 ALERT type 3 Indicator that the structure quality may be low
   0 ALERT type 4 Improvement, methodology, query or suggestion
   0 ALERT type 5 Informative message, check
```

It is advisable to attempt to resolve as many as possible of the alerts in all categories. Often the minor alerts point to easily fixed oversights, errors and omissions in your CIF or refinement strategy, so attention to these fine details can be worthwhile. In order to resolve some of the more serious problems it may be necessary to carry out additional measurements or structure refinements. However, the purpose of your study may justify the reported deviations and the more serious of these should normally be commented upon in the discussion or experimental section of a paper or in the "special_details" fields of the CIF. checkCIF was carefully designed to identify outliers and unusual parameters, but every test has its limitations and alerts that are not important in a particular case may appear. Conversely, the absence of alerts does not guarantee there are no aspects of the results needing attention. It is up to the individual to critically assess their own results and, if necessary, seek expert advice.

Publication of your CIF in IUCr journals

A basic structural check has been run on your CIF. These basic checks will be run on all CIFs submitted for publication in IUCr journals (*Acta Crystallographica*, *Journal of Applied Crystallography*, *Journal of Synchrotron Radiation*); however, if you intend to submit to *Acta Crystallographica Section C* or *E*, you should make sure that full publication checks are run on the final version of your CIF prior to submission.

Publication of your CIF in other journals

Please refer to the *Notes for Authors* of the relevant journal for any special instructions relating to CIF submission.

PLATON version of 29/01/2015; check.def file version of 29/01/2015

Figure S17. *Cont.*

Datablock shelxl - ellipsoid plot

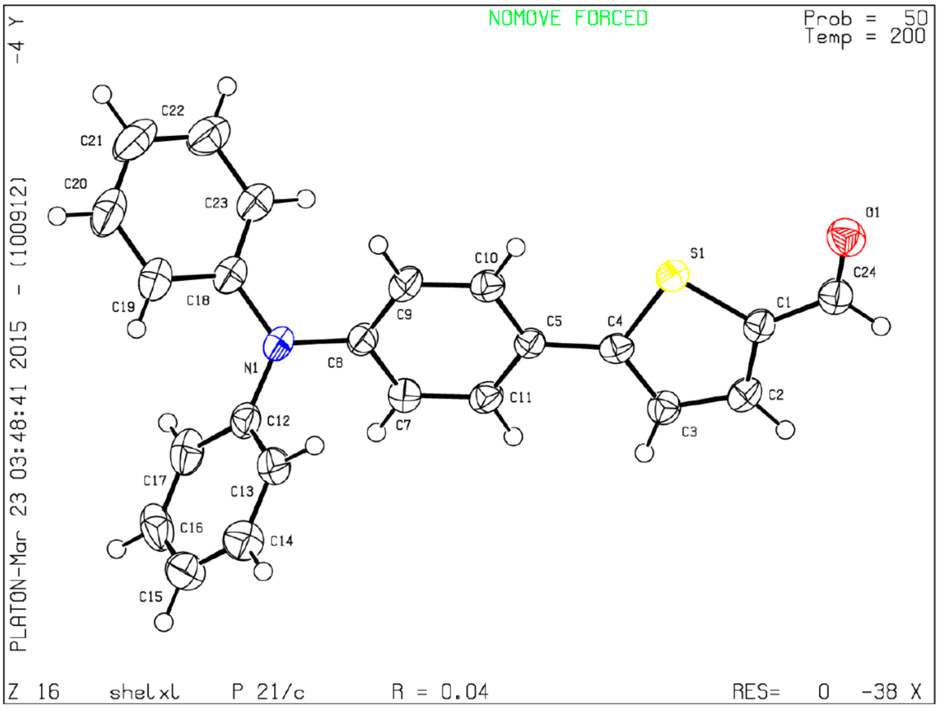

Figure S17. CIF of aldehyde (**1**).

Table S1. Fractional Atomic Coordinates (×10[4]) and Equivalent Isotropic Displacement Parameters (Å^2 × 10^3) for AS1. U_{eq} is defined as 1/3 of of the trace of the orthogonalised U_{IJ} tensor.

Atom	*x*	*y*	*z*	U(eq)
C1	5752.3(12)	4716(4)	3672.2(17)	30.3(6)
C2	5829.3(13)	2697(4)	3808.0(18)	35.1(6)
C3	5223.0(12)	1696(4)	3891.0(18)	32.6(6)
C4	4675.4(12)	2960(4)	3813.6(16)	28.2(6)
C5	3966.4(12)	2440(4)	3880.9(16)	27.0(6)
C7	3111.3(13)	−74(4)	4010.9(19)	35.4(7)
C8	2621.7(12)	1390(4)	4097.2(18)	32.0(6)
C9	2810.6(13)	3393(4)	4052.0(19)	36.7(7)
C10	3470.5(13)	3895(4)	3938.4(18)	33.4(6)
C11	3768.0(13)	449(4)	3913.5(18)	32.8(6)
C12	1931.8(13)	−686(4)	5047(2)	34.9(6)
C13	2309.2(13)	−395(4)	5974(2)	38.9(7)
C14	2289.5(16)	−1798(5)	6724(2)	52.8(8)
C15	1904.4(16)	−3467(5)	6581(3)	55.4(9)
C16	1535.0(16)	−3794(4)	5665(3)	55.1(9)
C17	1546.8(14)	−2421(4)	4886(2)	46.0(8)
C18	1375.7(13)	1828(4)	3883(2)	36.0(6)
C19	800.6(13)	1790(5)	4385(2)	45.0(7)
C20	228.3(15)	2830(5)	4004(3)	57.1(9)
C21	217.0(16)	3932(5)	3133(3)	61.4(10)
C22	782.5(15)	3966(5)	2634(2)	52.8(8)
C23	1354.5(14)	2913(4)	3002(2)	40.9(7)
C24	6278.6(14)	6183(4)	3599.4(19)	36.0(6)
N1	1964.5(10)	769(3)	4274.5(16)	38.5(6)
O1	6199.3(9)	7976(3)	3532.4(13)	43.4(5)
S1	4917.2(3)	5401.6(10)	3636.0(5)	32.8(2)

Table S2. Anisotropic Displacement Parameters (Å^2 × 10^3) for AS1. The Anisotropic displacement factor exponent takes the form: $-2\pi^2[h^2a^{*2}U_{11}+2hka^*b^*U_{12}+\ldots]$.

Atom	U_{11}	U_{22}	U_{33}	U_{23}	U_{13}	U_{12}
C1	27.6(14)	40.5(17)	22.5(13)	−1.1(11)	1.5(10)	1.3(12)
C2	30.0(14)	38.3(17)	36.8(15)	−2.4(12)	3.2(11)	6.9(12)
C3	31.3(14)	31.4(16)	35.1(15)	−2.0(11)	4.1(11)	1.3(12)
C4	33.2(14)	32.1(15)	18.8(13)	−1.1(11)	1.2(10)	3.0(12)
C5	26.8(13)	34.0(16)	20.1(12)	0.6(11)	2.2(10)	2.5(11)
C7	32.1(15)	34.9(16)	39.2(16)	1.7(12)	4.2(12)	−0.5(12)
C8	25.9(14)	39.4(17)	30.5(14)	3.3(12)	2.1(11)	2.2(12)
C9	28.2(14)	41.6(18)	40.3(16)	−0.9(13)	3.8(12)	7.7(12)
C10	34.3(15)	30.8(15)	34.8(15)	1.0(12)	3.7(11)	3.5(12)
C11	30.6(14)	34.3(16)	33.5(15)	1.4(12)	4.4(11)	7.2(12)
C12	26.2(14)	35.1(16)	44.3(16)	−0.8(13)	7.7(12)	2.2(12)
C13	34.5(16)	37.9(17)	44.3(17)	0.4(14)	4.1(12)	−5.1(13)
C14	49.5(19)	58(2)	51.3(19)	8.5(16)	7.3(14)	2.9(16)
C15	51(2)	49(2)	70(2)	16.5(17)	19.0(17)	6.8(16)
C16	45.0(19)	28.5(17)	97(3)	−4.8(18)	29.9(19)	−6.8(14)
C17	32.9(16)	47(2)	58.3(19)	−14.6(16)	6.7(13)	−4.7(13)
C18	28.7(15)	37.2(16)	41.1(16)	−6.8(13)	−0.3(12)	2.4(12)
C19	30.7(16)	49.4(19)	54.9(19)	−6.4(15)	4.8(13)	−0.5(13)
C20	30.1(16)	58(2)	83(2)	−10.1(19)	5.0(16)	4.4(15)
C21	38.0(19)	54(2)	87(3)	−3.1(19)	−15.8(17)	12.0(15)
C22	45(2)	48(2)	61(2)	−1.3(16)	−10.7(16)	5.2(15)
C23	34.1(15)	43.2(17)	44.0(17)	−4.7(14)	−1.5(12)	3.9(13)
C24	34.1(15)	43.5(18)	30.1(15)	−0.7(13)	2.7(11)	−0.6(13)
N1	23.0(12)	47.9(15)	43.7(14)	8.6(11)	0.7(10)	2.8(10)
O1	45.2(12)	41.5(13)	43.0(12)	0.3(9)	2.5(9)	−4.7(10)
S1	30.1(4)	34.4(4)	33.6(4)	3.1(3)	2.5(3)	3.0(3)

Table S3. Bond Lengths for **AS1**.

Atom	Atom	Length/Å	Atom	Atom	Length/Å
C1	C2	1.365(4)	C12	C13	1.392(4)
C1	C24	1.445(4)	C12	N1	1.428(3)
C1	S1	1.720(2)	C13	C14	1.379(4)
C2	C3	1.396(4)	C14	C15	1.353(4)
C3	C4	1.373(3)	C15	C16	1.377(5)
C4	C5	1.467(3)	C16	C17	1.392(4)
C4	S1	1.722(3)	C18	C23	1.384(4)
C5	C11	1.387(4)	C18	C19	1.395(4)
C5	C10	1.393(3)	C18	N1	1.417(3)
C7	C11	1.374(3)	C19	C20	1.381(4)
C7	C8	1.394(4)	C20	C21	1.379(5)
C8	C9	1.391(4)	C21	C22	1.376(5)
C8	N1	1.419(3)	C22	C23	1.380(4)
C9	C10	1.382(4)	C24	O1	1.208(3)
C12	C17	1.391(4)			

Table S4. Bond Angles for **AS1**.

Atom	Atom	Atom	Angle/°	Atom	Atom	Atom	Angle/°
C2	C1	C24	127.3(2)	C13	C12	N1	119.4(2)
C2	C1	S1	111.1(2)	C14	C13	C12	120.1(3)
C24	C1	S1	121.6(2)	C15	C14	C13	121.2(3)
C1	C2	C3	113.4(2)	C14	C15	C16	119.5(3)
C4	C3	C2	112.8(2)	C15	C16	C17	120.9(3)
C3	C4	C5	127.7(2)	C12	C17	C16	119.1(3)
C3	C4	S1	111.02(19)	C23	C18	C19	118.5(2)
C5	C4	S1	121.25(18)	C23	C18	N1	121.4(2)
C11	C5	C10	117.3(2)	C19	C18	N1	120.1(3)
C11	C5	C4	120.5(2)	C20	C19	C18	120.0(3)
C10	C5	C4	122.2(2)	C21	C20	C19	121.0(3)
C11	C7	C8	120.8(3)	C22	C21	C20	119.2(3)
C9	C8	C7	118.3(2)	C21	C22	C23	120.4(3)
C9	C8	N1	123.2(2)	C22	C23	C18	121.0(3)
C7	C8	N1	118.5(2)	O1	C24	C1	125.8(3)
C10	C9	C8	120.2(2)	C18	N1	C8	122.1(2)
C9	C10	C5	121.8(3)	C18	N1	C12	120.2(2)
C7	C11	C5	121.6(2)	C8	N1	C12	116.1(2)
C17	C12	C13	119.1(3)	C1	S1	C4	91.70(12)
C17	C12	N1	121.5(3)				

Table S5. Torsion Angles for **AS1**.

A	B	C	D	Angle/°	A	B	C	D	Angle/°
C24	C1	C2	C3	177.4(2)	C23	C18	C19	C20	−0.4(4)
S1	C1	C2	C3	−0.5(3)	N1	C18	C19	C20	179.8(3)
C1	C2	C3	C4	0.3(3)	C18	C19	C20	C21	−0.7(5)
C2	C3	C4	C5	−179.0(2)	C19	C20	C21	C22	0.9(5)
C2	C3	C4	S1	0.0(3)	C20	C21	C22	C23	−0.1(5)
C3	C4	C5	C11	−8.2(4)	C21	C22	C23	C18	−0.9(5)
S1	C4	C5	C11	172.89(18)	C19	C18	C23	C22	1.2(4)
C3	C4	C5	C10	170.3(2)	N1	C18	C23	C22	−179.0(3)
S1	C4	C5	C10	−8.7(3)	C2	C1	C24	O1	−175.8(3)
C11	C7	C8	C9	1.9(4)	S1	C1	C24	O1	1.8(4)
C11	C7	C8	N1	−175.1(2)	C23	C18	N1	C8	28.6(4)
C7	C8	C9	C10	−0.9(4)	C19	C18	N1	C8	−151.6(3)
N1	C8	C9	C10	176.0(2)	C23	C18	N1	C12	−165.9(2)
C8	C9	C10	C5	−1.0(4)	C19	C18	N1	C12	13.9(4)
C11	C5	C10	C9	1.8(4)	C9	C8	N1	C18	36.5(4)
C4	C5	C10	C9	−176.7(2)	C7	C8	N1	C18	−146.6(2)
C8	C7	C11	C5	−1.1(4)	C9	C8	N1	C12	−129.5(3)
C10	C5	C11	C7	−0.8(4)	C7	C8	N1	C12	47.3(3)
C4	C5	C11	C7	177.7(2)	C17	C12	N1	C18	64.0(3)
C17	C12	C13	C14	−1.1(4)	C13	C12	N1	C18	−118.0(3)
N1	C12	C13	C14	−179.2(2)	C17	C12	N1	C8	−129.7(3)
C12	C13	C14	C15	−0.4(5)	C13	C12	N1	C8	48.3(3)
C13	C14	C15	C16	1.3(5)	C2	C1	S1	C4	0.41(19)
C14	C15	C16	C17	−0.7(5)	C24	C1	S1	C4	−177.6(2)
C13	C12	C17	C16	1.7(4)	C3	C4	S1	C1	−0.26(19)
N1	C12	C17	C16	179.7(2)	C5	C4	S1	C1	178.83(19)
C15	C16	C17	C12	−0.8(4)					

Table S6. Fractional Atomic Coordinates (×10^4) and Equivalent Isotropic Displacement Parameters ($Å^2 \times 10^3$) for **AS2**. U_{eq} is defined as 1/3 of of the trace of the orthogonalised U_{IJ} tensor.

Atom	*x*	*y*	*z*	U(eq)
Cl	7297(2)	8374.8(13)	4936.0(9)	108.9(5)
Cl1	5445.2(18)	9262.2(9)	3228.2(8)	86.0(3)
Cl5	8022(3)	10543.6(11)	4045.7(12)	121.1(5)
S1	2804.4(8)	7696.3(5)	1111.9(4)	31.81(15)
N1	10844(3)	5259.8(17)	2946.1(17)	43.8(5)
N2	55(3)	9454(2)	2006.3(17)	50.1(6)
C1	-4197(3)	9256.6(19)	-540.2(17)	34.6(5)
C2	-3211(3)	9486.8(17)	235.6(15)	28.8(4)
C3	-4060(3)	10242.3(19)	770.1(16)	33.9(5)
C4	-1349(3)	8959.6(17)	485.5(15)	29.2(4)
C5	-550(3)	9224(2)	1335.1(17)	34.5(5)
C6	-381(3)	8274.0(18)	-23.9(16)	32.1(4)
C7	1397(3)	7689.8(18)	144.0(16)	31.1(4)
C8	2247(3)	7027.6(19)	-454.4(18)	37.2(5)
C9	3978(3)	6536.4(19)	-137.8(18)	36.7(5)
C10	4469(3)	6812.1(17)	715.9(15)	29.0(4)
C11	6161(3)	6445.9(17)	1281.1(16)	30.6(4)
C12	6292(3)	6674.8(19)	2197.7(17)	35.6(5)
C13	7829(4)	6300(2)	2744.1(18)	38.5(5)
C14	9294(3)	5680.9(19)	2394.7(17)	36.1(5)
C15	9185(3)	5474(2)	1468.7(18)	38.6(5)
C16	7644(3)	5854.4(19)	925.7(17)	34.9(5)
C17	11744(3)	4241.5(19)	2801.9(17)	36.7(5)
C18	13673(4)	4151(3)	2685(2)	52.1(7)
C19	14551(5)	3157(4)	2576(2)	70.1(11)
C20	13539(7)	2244(3)	2582(2)	74.2(12)
C21	11637(7)	2323(3)	2676(2)	67.7(10)
C22	10725(4)	3324(2)	2796(2)	48.7(6)
C23	11500(3)	5811(2)	3678.0(17)	36.2(5)
C24	11505(4)	6945(2)	3570.8(18)	39.9(5)
C25	12169(4)	7464(2)	4288(2)	47.2(6)
C26	12870(4)	6869(3)	5096(2)	52.8(7)
C27	12874(4)	5751(3)	5196(2)	52.5(7)
C28	12179(4)	5213(2)	4497(2)	43.7(6)
C29	7506(6)	9217(3)	3893(3)	66.2(9)

Table S7. Anisotropic Displacement Parameters (Å^2 × 10^3) for **AS2**. The Anisotropic displacement factor exponent takes the form: $-2\pi^2[h^2a^{*2}U_{11}+2hka^*b^*U_{12}+\ldots]$.

**Atom**	**U_{11}**	**U_{22}**	**U_{33}**	**U_{23}**	**U_{13}**	**U_{12}**
Cl	102.2(9)	135.6(12)	77.8(8)	28.8(7)	13.5(7)	−5.0(8)
Cl1	102.7(8)	76.1(6)	78.3(7)	−0.5(5)	1.5(6)	−16.6(6)
Cl5	156.3(14)	89.1(8)	131.6(13)	−53.0(9)	17.4(10)	−39.3(9)
S1	31.6(3)	35.2(3)	28.4(3)	−6.3(2)	−4.1(2)	4.4(2)
N1	45.1(11)	39.0(11)	48.6(13)	−17.1(9)	−20.6(10)	11.3(9)
N2	47.1(12)	65.4(15)	38.6(12)	−17.0(11)	−9(1)	12.4(11)
C1	35.3(11)	37.7(11)	31.7(11)	−10.9(9)	−4.2(9)	5.3(9)
C2	29.1(9)	28.7(9)	27.9(10)	−1.4(8)	−1.8(8)	0.8(7)
C3	35.3(11)	37.6(11)	29.4(11)	−8.6(9)	−6.4(9)	2.4(9)
C4	30.3(9)	29.2(9)	27.1(10)	−0.7(8)	−4.0(8)	−0.8(8)
C5	33.1(10)	38.6(11)	31.0(11)	−4.4(9)	−1.7(8)	4.8(9)
C6	32.2(10)	32.6(10)	31.7(11)	−5.9(8)	−6.1(8)	1.3(8)
C7	31.9(10)	30.9(10)	30.3(11)	−4.9(8)	−5.5(8)	1.7(8)
C8	40.3(12)	36.8(11)	35.8(12)	−11.5(9)	−11(1)	3.7(9)
C9	37.4(11)	35.5(11)	38.5(12)	−13.7(9)	−7.5(9)	7.1(9)
C10	30.9(10)	26.8(9)	29.1(10)	−3.4(8)	−4.2(8)	1.1(7)
C11	32.7(10)	27.7(9)	31.7(11)	−4.1(8)	−3.7(8)	−2.3(8)
C12	35.4(11)	35.9(11)	36.1(12)	−10.0(9)	−6.2(9)	6.7(9)
C13	42.4(12)	41.0(12)	33.1(12)	−11.2(10)	−9(1)	5.5(10)
C14	36.7(11)	34.3(11)	37.9(12)	−9.2(9)	−9.6(9)	2.4(9)
C15	35.6(11)	39.2(12)	42.3(13)	−14.6(10)	−7.5(10)	8.4(9)
C16	37.3(11)	36.7(11)	31.5(11)	−9.3(9)	−6.3(9)	2.6(9)
C17	41.2(12)	34.6(11)	33.8(12)	−6.5(9)	−8.0(9)	6.7(9)
C18	42.6(14)	67.9(19)	45.4(16)	−9.4(14)	−4.4(12)	4.7(13)
C19	58.9(19)	102(3)	48.4(18)	−22.2(19)	−6.5(15)	37(2)
C20	108(3)	67(2)	45.2(18)	−20.7(16)	−21.9(19)	47(2)
C21	120(3)	37.2(14)	46.0(17)	−7.0(12)	−14.3(19)	−2.0(17)
C22	57.4(16)	41.5(13)	47.6(16)	−5.6(11)	−3.5(13)	−5.2(12)
C23	36.5(11)	37.2(11)	35.8(12)	−9.9(9)	−7.0(9)	1.5(9)
C24	44.3(13)	40.0(12)	36.0(12)	−7.9(10)	−2.4(10)	−1.7(10)
C25	52.8(15)	45.8(14)	46.3(15)	−16.3(12)	0.9(12)	−8.1(12)
C26	51.9(16)	66.7(19)	44.9(16)	−24.5(14)	−7.0(12)	−8.0(14)
C27	51.5(15)	68.7(19)	36.6(14)	−6.9(13)	−10.7(12)	2.5(14)
C28	44.6(13)	42.2(13)	43.5(14)	−3.5(11)	−10.9(11)	−0.1(11)
C29	82(2)	59.1(19)	57(2)	−10.7(16)	19.0(17)	1.0(17)

Table S8. Bond Lengths for **AS2**.

Atom	Atom	Length/Å	Atom	Atom	Length/Å
Cl	C29	1.733(4)	C10	C11	1.480(3)
Cl1	C29	1.758(4)	C11	C16	1.389(3)
Cl5	C29	1.738(4)	C11	C12	1.397(3)
S1	C10	1.716(2)	C12	C13	1.380(3)
S1	C7	1.730(2)	C13	C14	1.394(3)
N1	C14	1.405(3)	C14	C15	1.401(3)
N1	C17	1.417(3)	C15	C16	1.382(3)
N1	C23	1.425(3)	C17	C22	1.381(4)
N2	C5	1.140(3)	C17	C18	1.390(4)
C1	C3 [1]	1.383(3)	C18	C19	1.371(5)
C1	C2	1.397(3)	C19	C20	1.372(6)
C2	C3	1.397(3)	C20	C21	1.366(6)
C2	C4	1.475(3)	C21	C22	1.393(4)
C3	C1 [1]	1.383(3)	C23	C24	1.386(3)
C4	C6	1.352(3)	C23	C28	1.390(4)
C4	C5	1.441(3)	C24	C25	1.386(4)
C6	C7	1.432(3)	C25	C26	1.380(4)
C7	C8	1.381(3)	C26	C27	1.367(5)
C8	C9	1.400(3)	C27	C28	1.388(4)
C9	C10	1.376(3)			

[1] 1 – X, 2 – Y, –Z.

Table S9. Bond Angles for **AS2**.

Atom	Atom	Atom	Angle/°	Atom	Atom	Atom	Angle/°
C10	S1	C7	92.58(10)	C12	C13	C14	120.7(2)
C14	N1	C17	119.77(19)	C13	C14	C15	118.2(2)
C14	N1	C23	121.57(19)	C13	C14	N1	121.8(2)
C17	N1	C23	118.61(19)	C15	C14	N1	120.0(2)
C3 [1]	C1	C2	121.3(2)	C16	C15	C14	120.7(2)
C1	C2	C3	117.25(19)	C15	C16	C11	121.2(2)
C1	C2	C4	121.8(2)	C22	C17	C18	119.2(3)
C3	C2	C4	120.97(19)	C22	C17	N1	120.5(2)
C1 [1]	C3	C2	121.4(2)	C18	C17	N1	120.2(2)
C6	C4	C5	119.67(19)	C19	C18	C17	120.2(3)
C6	C4	C2	124.7(2)	C18	C19	C20	120.5(3)
C5	C4	C2	115.61(19)	C21	C20	C19	120.1(3)
N2	C5	C4	178.5(3)	C20	C21	C22	120.2(3)
C4	C6	C7	130.6(2)	C17	C22	C21	119.8(3)
C8	C7	C6	124.4(2)	C24	C23	C28	119.6(2)
C8	C7	S1	109.62(16)	C24	C23	N1	120.4(2)
C6	C7	S1	125.96(17)	C28	C23	N1	120.0(2)
C7	C8	C9	114.1(2)	C23	C24	C25	119.4(2)
C10	C9	C8	112.6(2)	C26	C25	C24	121.0(3)
C9	C10	C11	128.6(2)	C27	C26	C25	119.4(2)
C9	C10	S1	111.14(16)	C26	C27	C28	120.7(3)
C11	C10	S1	120.25(16)	C27	C28	C23	119.9(3)
C16	C11	C12	118.1(2)	Cl	C29	Cl5	112.8(2)
C16	C11	C10	121.4(2)	Cl	C29	Cl1	110.7(2)
C12	C11	C10	120.5(2)	Cl5	C29	Cl1	109.2(2)
C13	C12	C11	121.1(2)				

[1] 1 – X, 2 – Y, –Z.

Table S10. Torsion Angles for **AS2**.

A	B	C	D	Angle/°	A	B	C	D	Angle/°
$C3^1$	C1	C2	C3	0.1(4)	C17	N1	C14	C15	−32.7(4)
$C3^1$	C1	C2	C4	−179.8(2)	C23	N1	C14	C15	149.9(3)
C1	C2	C3	$C1^1$	−0.1(4)	C13	C14	C15	C16	−1.4(4)
C4	C2	C3	$C1^1$	179.8(2)	N1	C14	C15	C16	178.0(2)
C1	C2	C4	C6	4.2(4)	C14	C15	C16	C11	−0.3(4)
C3	C2	C4	C6	−175.7(2)	C12	C11	C16	C15	1.9(4)
C1	C2	C4	C5	−176.3(2)	C10	C11	C16	C15	−177.2(2)
C3	C2	C4	C5	3.8(3)	C14	N1	C17	C22	−52.5(4)
C5	C4	C6	C7	2.3(4)	C23	N1	C17	C22	125.1(3)
C2	C4	C6	C7	−178.3(2)	C14	N1	C17	C18	128.9(3)
C4	C6	C7	C8	−178.5(3)	C23	N1	C17	C18	−53.5(4)
C4	C6	C7	S1	1.6(4)	C22	C17	C18	C19	−0.8(4)
C10	S1	C7	C8	−0.8(2)	N1	C17	C18	C19	177.8(3)
C10	S1	C7	C6	179.2(2)	C17	C18	C19	C20	−0.1(5)
C6	C7	C8	C9	−179.6(2)	C18	C19	C20	C21	1.5(5)
S1	C7	C8	C9	0.3(3)	C19	C20	C21	C22	−2.1(5)
C7	C8	C9	C10	0.5(3)	C18	C17	C22	C21	0.2(4)
C8	C9	C10	C11	178.8(2)	N1	C17	C22	C21	−178.4(3)
C8	C9	C10	S1	−1.0(3)	C20	C21	C22	C17	1.3(5)
C7	S1	C10	C9	1.03(19)	C14	N1	C23	C24	−38.4(4)
C7	S1	C10	C11	−178.84(19)	C17	N1	C23	C24	144.1(3)
C9	C10	C11	C16	9.1(4)	C14	N1	C23	C28	143.1(3)
S1	C10	C11	C16	−171.08(18)	C17	N1	C23	C28	−34.4(4)
C9	C10	C11	C12	−170.0(2)	C28	C23	C24	C25	−0.8(4)
S1	C10	C11	C12	9.9(3)	N1	C23	C24	C25	−179.3(3)
C16	C11	C12	C13	−1.7(4)	C23	C24	C25	C26	1.8(4)
C10	C11	C12	C13	177.4(2)	C24	C25	C26	C27	−1.3(5)
C11	C12	C13	C14	0.0(4)	C25	C26	C27	C28	−0.1(5)
C12	C13	C14	C15	1.6(4)	C26	C27	C28	C23	1.1(5)
C12	C13	C14	N1	−177.9(2)	C24	C23	C28	C27	−0.7(4)
C17	N1	C14	C13	146.8(3)	N1	C23	C28	C27	177.9(3)
C23	N1	C14	C13	−30.7(4)					
